# Neuronal differentiation is associated with a redox-regulated increase of copper flow to the secretory pathway

**DOI:** 10.1038/ncomms10640

**Published:** 2016-02-16

**Authors:** Yuta Hatori, Ye Yan, Katharina Schmidt, Eri Furukawa, Nesrin M. Hasan, Nan Yang, Chin-Nung Liu, Shanthini Sockanathan, Svetlana Lutsenko

**Affiliations:** 1 Department of Physiology, Johns Hopkins University, School of Medicine, 725 N. Wolfe street, Baltimore, 21205 Maryland, USA; 2 Department of Neuroscience, Johns Hopkins University, School of Medicine, 725 N. Wolfe street, Baltimore, Maryland 21205, USA

## Abstract

Brain development requires a fine-tuned copper homoeostasis. Copper deficiency or excess results in severe neuro-pathologies. We demonstrate that upon neuronal differentiation, cellular demand for copper increases, especially within the secretory pathway. Copper flow to this compartment is facilitated through transcriptional and metabolic regulation. Quantitative real-time imaging revealed a gradual change in the oxidation state of cytosolic glutathione upon neuronal differentiation. Transition from a broad range of redox states to a uniformly reducing cytosol facilitates reduction of the copper chaperone Atox1, liberating its metal-binding site. Concomitantly, expression of Atox1 and its partner, a copper transporter ATP7A, is upregulated. These events produce a higher flux of copper through the secretory pathway that balances copper in the cytosol and increases supply of the cofactor to copper-dependent enzymes, expression of which is elevated in differentiated neurons. Direct link between glutathione oxidation and copper compartmentalization allows for rapid metabolic adjustments essential for normal neuronal function.

## Abstract (web summary)

Differentiating neurons have an increased requirement for copper than their precursors, but the mechanism of altered copper homoeostasis is not known. Here, Hatori *et al*. show that neuronal differentiation is accompanied by an increased flux of copper through the secretory pathway, increasing supply to copper-dependent enzymes.

## Graphical abstract

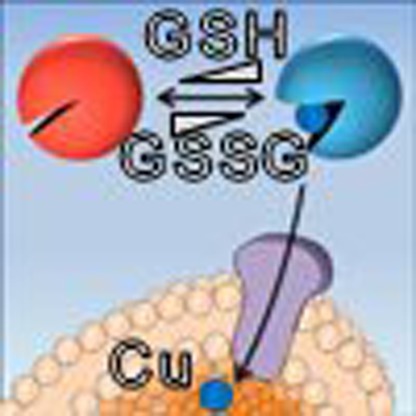

Neuronal cells require copper for their neurochemical activities as well as general metabolism. The ability of copper to cycle between the two oxidation states (Cu^+^ and Cu^2+^) is utilized by various enzymes that carry out biochemical reactions essential for brain development and function. The housekeeping copper-dependent enzymes include cytochrome c oxidase, which is involved in electron transfer and ATP production in mitochondria, and superoxide dismutases (SOD1 and SOD3), which are responsible for detoxification of reactive oxygen species (ROS) in the cytosol and at the cell surface, respectively. Copper-dependent enzymes that contribute to functional identity of specific neurons include dopamine-β-hydroxylase (DBH), which performs the key step in the biosynthesis of norepinephrine, and peptidyl-glycine-α-monooxygenase (PAM), which is responsible for the production of all amidated neuropeptides^1^.

The copper-requiring proteins are located in different cellular compartments; this property necessitates a timely delivery of copper to these compartments for functional maturation of resident enzymes. It has been established that after entering cells copper is escorted to specific destinations by small proteins called copper chaperones^2^. The chaperone for SOD, CCS, delivers copper to SOD1 in the cytosol, whereas a complex set of chaperones including Cox11, Cox17, SCO1 and SCO2 mediates copper transfer into mitochondria and incorporation of copper into cytochrome c oxidase. The cytosolic chaperone Atox1 shuttles copper to the copper transporters ATP7A and ATP7B located in the secretory pathway^2^,^3^,^4^. Using the energy of ATP hydrolysis, ATP7A and ATP7B transport cytosolic copper into the lumen of *trans*-Golgi network (TGN) and various vesicles to activate PAM, DBH, SOD3 and other copper-dependent enzymes.

Although identities of metallochaperones have been established, it remains unclear how cells regulate copper flow to different cellular compartments to meet the metabolic demands for copper in these locations. Biochemical studies led to the suggestion that copper distribution between proteins is driven by relative abundance of copper-binding molecules and differences in their affinity for copper^5^. This basic principle is likely to govern copper distribution in the cytosol under steady-state conditions. The mechanisms through which cells make changes in copper delivery to any given compartment remain unclear. Yet, timely adjustment of intracellular copper fluxes is potentially crucial for the development of unique neurochemical characteristics of neurons. Upon transition from a proliferative phase to a differentiated state, neuronal cells undergo significant metabolic restructuring defined by changes in metabolome^6^ and protein-expression profiles^7^. Differentiated neurons actively synthesize neurotransmitters and neuropeptides within the secretory pathway. These processes require copper^8^. We hypothesized that neuronal differentiation is likely to involve adjustment of cellular copper homoeostasis, and, in the current study, we directly tested this hypothesis.

Transcriptional control is compatible with a long-term maintenance of cellular copper homoeostasis^9^. However, changes in protein expression are too slow to provide rapid adjustment of copper delivery to distinct subcellular locations, which may require post-translational and/or metabolic controls. We have previously found that the copper chaperone Atox1 can be reversibly oxidized within a physiologically relevant range of redox potentials^10^,^11^, and hypothesized that such reversible oxidation might modulate intracellular copper fluxes. Here, using *in vivo* and cell culture models, we investigated how differentiating neurons regulate intracellular copper distribution and whether this regulation involves redox modulation of copper-handling proteins. Our experiments revealed two distinct mechanisms through which cells adjust intracellular copper fluxes and uncovered a tight link between the cellular metal compartmentalization and redox homeostasis. Our results suggest that changes in the redox status of cytosolic glutathione may have significant effect on the metabolic activity of secretory pathway by altering maturation and function of the resident copper-dependent enzymes.

## Results

### Differentiated motor neurons have higher levels of PAM

**Figure 1:**
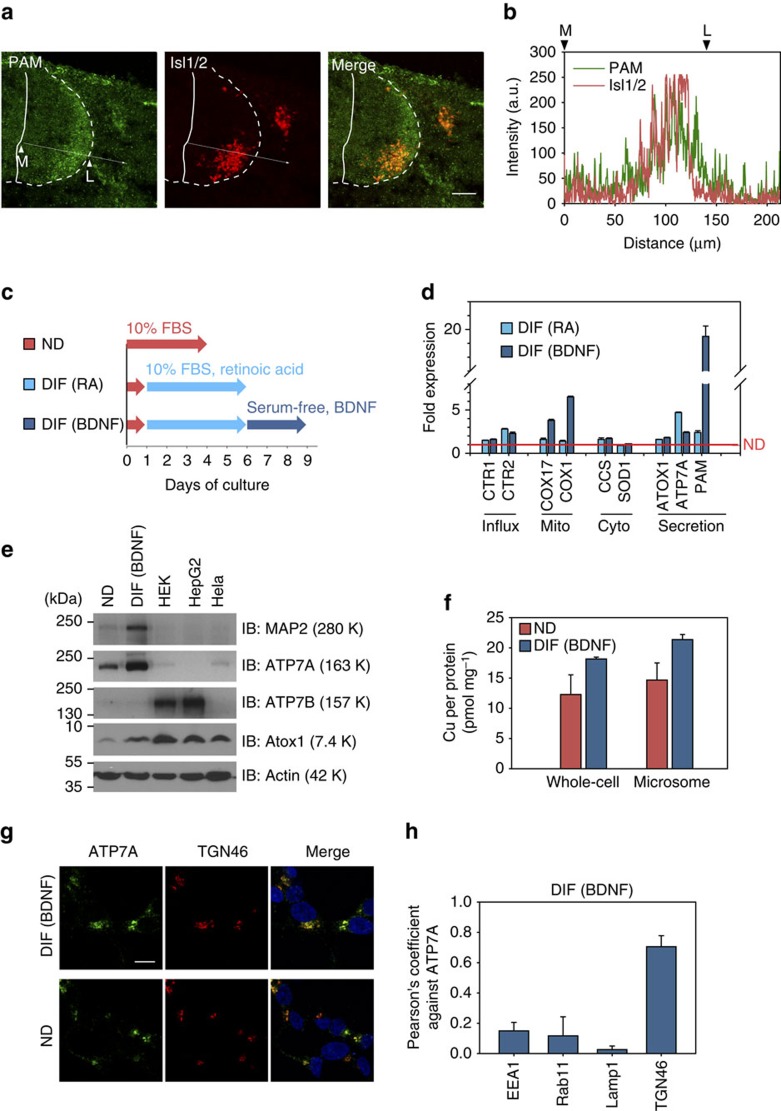
Requirement for copper is increased in the secretory pathway upon neuronal differentiation. (**a**) Immunostaining of PAM (green) in a chicken spinal cord (HH stage 20 embryo). Traverse sections of chick embryos were analysed. Differentiated cells in marginal zones were identified by postmitotic marker isl1/2 (red). Scale bar, 50 μm. (**b**) Intensity profile along the line indicated by the arrow in **a** shows overlap between PAM and Isl1/2 expression. Medial and lateral borders are represented as M and L, respevtively. (**c**) Schematic of differentiation of SH-SY5Y cells by sequential treatments with retinoic acid (DIF RA) and BDNF (DIF BDNF). (**d**) Differentiation is associated with upregulation of genes involved in copper fluxes to mitochondria and, especially, the secretory pathway. The mRNA levels were determined by ΔΔCt analysis using GAPDH as a reference gene and normalized to non-differentiated cells (red line). Each value is presented as mean±s.d. (*n*=3). (**e**) Protein levels for ATP7A and Atox1 increase upon differentiation. Equal amount of protein was loaded to each lane. Neuronal differentiation was verified by upregulation of MAP2. (**f**) Cellular copper content in differentiated SH-SY5Y cells (BDNF) is higher than in non-differentiated (ND) cells. Copper amounts were determined by atomic absorption and normalized to protein amounts. Data from three independent measurements. (**g**,**h**) ATP7A is localized within TGN and vesicular structures which are distinct from endosomal or lysosomal compartments. Coimmunostaining with TGN was shown as a representative image (see Supplementary Fig. 2a for other images). Nucleus was stained with DAPI (blue). Scale bar, 10 μm. Colocalization of ATP7A and various markers were evaluated using Pearson's product-moment coefficients. Three replicate samples were prepared and analysed. Each value is presented as mean±s.d. (*n*=3).

To test whether copper utilization changes upon neuronal differentiation we used two different experimental models. For the *in vivo* model, we have chosen the developing chick embryonic spinal cord. At Hamburger Hamilton stage (HH stage) 20–21, motor neuron progenitors differentiate into postmitotic motor neurons^12^. The progression of differentiation correlates with the position of cell bodies along the medial–lateral axis of the spinal cord^12^,^13^. Actively cycling neuronal progenitors reside medially within the ventricular zone, whereas the cell bodies of postmitotic motor neurons—visualized by the postmitotic motor neuron marker Isl1/2—are located laterally^13^ (Fig. 1a). The spinal cord is rich in neuropeptides including substance P^14^,^15^, which is involved in chemoattraction of migrating cells. Functional maturation of substance P and other neuropeptides requires a copper-dependent enzyme peptidylglycine-α-amidating monooxygenase (PAM)^8^. Immunohistochemistry of chick spinal cords revealed that PAM is more abundant in the lateral region compared to the medial zone (Fig. 1a). Consistent with this observation, differentiated motor neurons (indicated by the marker Isl1/2) have higher levels of PAM than neuroprogenitors, as evidenced by colocalization of PAM and Isl1/2 (Fig. 1b). Higher abundance of PAM in differentiated motor neurons indicates an increased demand for copper in the secretory pathway to accommodate PAM biosynthesis.

### Expression of copper-proteins changes upon differentiation

To verify our *in vivo* observations and quantitatively measure levels of PAM and copper in differentiating neurons, we utilized a cultured cell model. Neuroblastoma SH-SY5Y cells were differentiated by sequential treatment with retinoic acid (RA) and brain-derived neurotrophic factor (BDNF)^16^ as shown in Fig. 1c. RA induces cell differentiation, as detected by the appearance of neuritic processes. Subsequent BDNF treatment in the absence of serum yields a homogeneous population of cells with a neuron-like phenotype^16^. Cell differentiation was further verified by expression of the neuronal marker microtubule-associated protein 2. Cell lysates were then used to determine copper content and the mRNA levels for PAM and proteins involved in copper transport and utilization.

Copper delivery to the secretory pathway is mediated by Atox1 and the downstream transporters ATP7A and ATP7B (ref. 4). We found Atox1 and ATP7A to be upregulated upon cell differentiation, whereas ATP7B was not (Fig. 1d and Supplementary Fig. 1a). The change in protein abundance was verified by western blotting and compared with other cell types (Fig. 1e). ATP7A levels in the non-differentiated SH-SY5Y cells were significantly higher than in other cells including HEK293, HeLa and HepG2 (Fig. 1e and Supplementary Fig. 1a) suggesting its functional importance in neuronal cells. The ATP7A abundance was further increased by RA (a trigger of neuronal differentiation) and remained steady during BDNF treatment (Supplementary Fig. 1b). Treatment of non-differentiating HEK293 cells with RA did not affect ATP7A levels (Supplementary Fig. 1c,d). These results suggested that ATP7A plays an important role in neuronal cells, especially post-differentiation (see Supplementary Fig. 1e,f for supporting evidence).

When measuring levels of copper-utilizing proteins, we observed significant differences between cell compartments. We detected a 19-fold increase in PAM mRNA which receives copper cofactor in the secretory pathway, the predicted location for ATP7A (Fig. 1d). Another important copper-dependent enzyme, DBH, was also upregulated in response to RA and was significantly higher in differentiated cells (Supplementary Fig. 1f,g) The mitochondria copper chaperone COX17 and subunit 1 of cytochrome C oxidase (COX1) were upregulated by 3.8-fold and 6.5-fold, respectively. This observation complemented a recent report showing that differentiation of SH-SY5Y cells increases cellular respiration^17^. In contrast, the cytosolic SOD1 and its copper chaperone CCS did not show significant changes at the mRNA levels.

Increased production of copper-dependent enzymes in the secretory pathway and mitochondria implied that differentiated cells require more copper in these compartments, whereas the demand for copper in the cytosol appeared unchanged. Measurements of copper in cell lysates confirmed that copper level in differentiated cells was higher than in non-differentiated cells (18.1±0.3 and 12.3±3.2 pmol per mg of protein, respectively, Fig. 1f). Copper levels in the microsomal fractions (containing TGN and secretory vesicles) were also higher in differentiated cells (21.4±0.8 pmol per mg compared with 14.7±2.8 pmol per mg in non-differentiated cells). The mRNA levels of copper uptake transporters CTR1 and CTR2 were increased (1.6-fold and 2.3-fold) upon differentiation (Fig. 1d), pointing to a potential source of elevated copper. Altogether, our results suggest that neuronal differentiation is associated with increased utilization of copper in the secretory pathway (and possibly mitochondria) and upregulated expression of proteins involved in copper uptake and transport to these compartments.

### Localization of ATP7A and Atox1 in SH-SY5Y cells

Previous studies demonstrated that increase in the cytosolic copper triggers trafficking of ATP7A from the TGN to vesicles^18^,^19^,^20^,^21^,^22^. Since total copper was higher in differentiated cells we tested whether localization of ATP7A may change with neuronal differentiation. Immunostaining of the non-differentiated and differentiated cells revealed that in both conditions, ATP7A had similar distribution patterns (Fig. 1g). It was detected at the periphery of nucleus (where it colocalized with the TGN marker TGN46; Pearson's product-moment coefficient *P*=0.71; Fig. 1h), and in vesicles distributed throughout the cell body (Supplementary Fig. 2a,c). Specificity of immunostaining was confirmed using transfection with ATP7A-targeted siRNA (Supplementary Fig. 2b). Similar intracellular distribution of ATP7A in differentiated and non-differentiated cells (despite difference in total copper) suggested that in differentiated cells extra copper was sequestered/compartmentalized and not available to stimulate ATP7A trafficking. The general ability of ATP7A to traffic was verified by treating cells with exogenous copper, which decreased the perinuclear signal and increased vesicular staining, which is indicative of trafficking (Supplementary Fig. 2c,d).

Atox1 is thought to be present mostly in the cytosol where it transiently binds copper and mediates subsequent metal transfer to ATP7A and ATP7B (refs 2, 4). Atox1 was also shown to translocate into the nucleus of mouse embryonic fibroblast where it may function as a transcription factor^23^ or copper importer^24^. We determined distribution of endogenous Atox1 by subcellular fractionation followed by western blotting (Supplementary Fig. 3a) and observed several bands. The 7.4-kDa band in a soluble fraction (cytosol) corresponded to the expected molecular weight of Atox1 (7.4 kDa) and 12–15 kDa bands in pellet fraction (nucleus) could have represented an Atox1 dimer. Treatment with siRNA^*Atox1*^ diminished the intensity of 7.4 kDa band but did not affect the 12–15-kDa bands, indicating that the latter bands are results of non-specific staining. Thus, at steady state, the majority of endogenous Atox1 is in the cytosol where it serves as a copper donor to ATP7A; this distribution does not change upon differentiation (Supplementary Fig. 3b).

### Glutathione oxidation decreases upon motor neuron maturation

**Figure 2:**
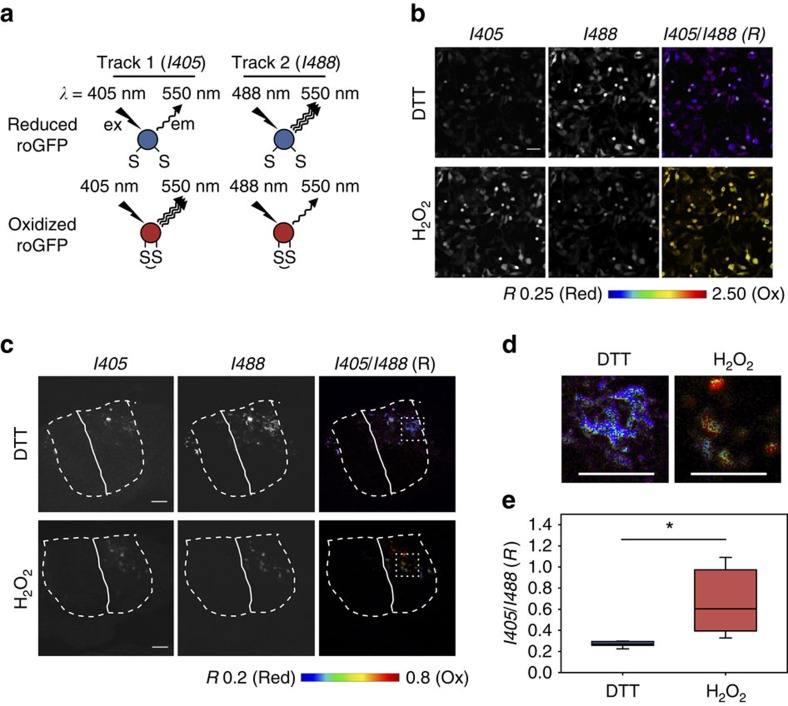
*In vivo* measurements of the redox status of cytosolic glutathione pair in neuronal cells of chicken spinal cord using Grx1-roGFP2. (**a**) The principle of the assay using Grx1-roGFP2. (**b**) HEK293 cells were transfected with pCDNA5/Grx1-roGFP2 and the redox response was tested by sequential treatments with 500 μM H_2_O_2_ and 2 mM DTT. *R* value (*I405*/*I488* ratio) is represented in a false-colour scale. Pixels with intensity higher than 127 (8-bit image) in either *I405* or *I488* are masked. Scale bars, 50 μm. (**c**) Grx1-roGFP2 was expressed in embryonic chick spinal cords by *inovo* electroporation and its redox response was verified by treating embryos with either 20 mM DTT or 20 mM H_2_O_2_ for 10 min before tissue fixation. The boundaries of the spinal cord are marked by dashed white line. The midline is marked by a solid white line. *R* value is shown in pseudo-colour scale. Scale bar, 50 μm. Technical details are documented in Supplementary Methods and Supplementary Figs 4–6. (**d**) Zoomed images of the areas boxed in **c**. Scale bar, 50 μm. (**e**) *R* values for multiple cells in **c** were measured and represented by box plots; the lower and upper boundaries of the box indicates the 25th and 75th percentiles, while whiskers and dots represent the 10th/90th and 5th/95th percentiles. A horizontal line within the box marks the median. Data are presented as mean±s.d.; 0.67±0.29 (H_2_O_2_, *n*=15), 0.27±0.03 (DTT, *n*=15). *Student's *t*-test, significant at *P*<0.001.

Differentiation of motor neurons involves a redox-dependent activation of glycerophosphodiester phosphodiesterase 2 (ref. 25), illustrating the impact of redox physiology on neuronal development. We have previously found that the oxidation state of Atox1 can be influenced by the redox status of a glutathione pair (GSH:GSSG (oxidized glutathione))^10^. Consequently, we hypothesized that the oxidation state of Atox1 may change during neuronal differentiation if the redox equilibrium of cytosolic glutathione is altered. To quantitatively measure the redox status of cytosolic glutathione upon differentiation we used the GSH:GSSG sensor based on a redox-sensitive green fluorescent protein (GFP) fused with glutaredoxin 1 (Grx1-roGFP2) (ref. 26). The sensor has two Cys residues that form a reversible disulfide bond depending on the oxidation state of cytosolic glutathione; formation of the disulfide bond alters the excitation spectrum of the sensor (Fig. 2a). The ratio of emission intensities following excitation at 405 and 488 nm (*R* value) reports the degree of sensor oxidation; a higher *R* value corresponds to a more oxidized state of glutathione (Fig. 2b and Supplementary Fig. 4).

Grx1-roGFP2 has not been previously used in vertebrate tissues. Consequently, we first tested whether the sensor can be used to quantitatively measure the redox status of cytosolic glutathione in a developing chick spinal cord. The pCDNA5/Grx1-roGFP2 plasmid was electroporated unilaterally into the spinal cords of chicken embryos at HH stage 12–14, before the initiation of motor neuron differentiation^27^. The Grx1-roGFP2 signal was observed specifically in the electroporated side of the spinal cord (right side; Fig. 2c and Supplementary Fig. 5). Soaking embryos in buffer containing either 20 mM DTT or 20 mM H_2_O_2_ yielded easily measurable difference in the Grx1-roGFP2 signal, which established the dynamic range for redox response of the sensor in the spinal cord (Fig. 2c–e). We verified that the *R* value was independent of the expression level of Grx1-roGFP2 (Supplementary Fig. 7), which is in agreement with a previous report^26^.

**Figure 3:**
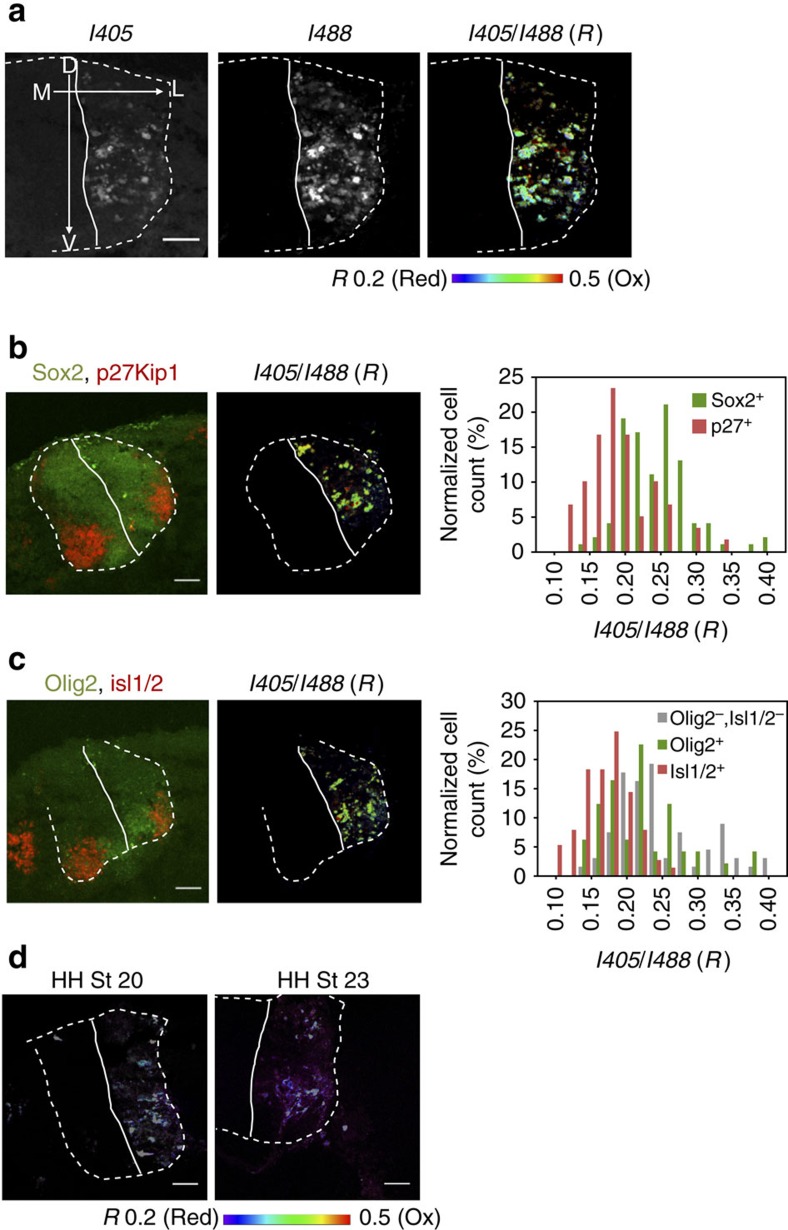
Redox status of cytosolic glutathione within neurons of chicken spinal cord. (**a**) Representative Grx1-roGFP2 image illustrates variation of redox states in neuronal cell population within a spinal cord. The *R* values (0.20–0.50) are shown with the pseudo-colour scale that corresponds to 0 to 29% oxidation of the sensor. Scale bars, 50 μm. (**b**) Distribution of *R* values in different cell populations. The sections were stained for Sox2 and p27Kip1 which define the regions of actively cycling cells (Sox2^+^) and differentiated cells (p27Kip1). Scale bar, 50 μm. (higher magnification is shown in Supplementary Fig. 9a). The ratio I405/I488 (*R* value) for Sox2^+^ (0.24±0.06; *n*=102) is significantly higher than in p27Kip1^+^ (0.18±0.05, *n*=60) regions (Student's *t*-test, *P*<0.001). (**c**) The sections were stained for Olig2 and Isl1/2 which define cycling motor neuron progenitors and postmitotic motor neurons, respectively. Scale bar, 50 μm. The *R* values for Olig2^−^:Isl1/2^−^ (0.25±0.07; *n*=68), Olig2^+^:Isl1/2^−^(0.23±0.09, *n*=49) and Olig2^−^:Isl1/2^+^ (0.16±0.03, *n*=77) regions. See Supplementary Fig. 9b for magnified view. Differences between Olig2^−^/Isl1/2^+^ from other regions are significant at *P*<0.001. There is no statistically significant difference between Olig2^−^:Isl1/2^−^ and Olig2^+^:Isl1/2^−^ regions (*P*=0.06). (**d**) Cytosolic glutathione in neuronal cells is more reduced in embryos at the later development stage. Embryos at HH stage 23 were compared with the stage 20 embryos (see Supplementary Fig. 8b and **c** for full information). Scale bar, 50 μm.

Using the developed protocol, we analysed eight embryos in two independent experiments to determine whether redox balance changes along with neuronal differentiation. In all embryos, Grx1-roGFP2 reported redox gradients along the medial–lateral axis of the spinal cord (Fig. 3a and Supplementary Fig. 8a). Expression of Sox2 and p27Kip1 defines the regions of actively cycling progenitors (Sox2^+^) and differentiated postmitotic neurons (p27Kip1^+^), respectively^25^. The mean *R* value for p27Kip1 positive cells (0.18±0.05) was lower than for Sox2^+^ region (0.24±0.06), suggesting that differentiated postmitotic cells have a larger fraction of reduced glutathione than cycling cells (Fig. 3b and Supplementary Fig. 9a). Similarly, the marginal zone containing postmitotic Isl1/2-expressing motor neurons showed more reduced environment (*R*=0.16±0.03) compared with the ventral Olig2^+^ positive region (*R*=0.23±0.09) or dorsal (Olig2^−^:Isl1/2^−^) ventricular zone (*R*=0.25±0.07), where cycling progenitor cells are located (Fig. 3c and Supplementary Fig. 9b). We confirmed that the immunostained sections had redox patterns similar to the non-stained samples (Supplementary Fig. 9c–e), excluding the possibility that staining interfered with the roGFP signal.

We further analysed embryos at a later stage (HH stage 23) when motor neuron differentiation within the spinal cord is nearing completion. Unlike HH stage 20 embryos, the redox status of glutathione (GSH:GSSG) was uniform and highly reduced throughout the spinal cord (Fig. 3d). Similar result was obtained for another section in a different axial position (Supplementary Fig. 8c). These data demonstrated that in addition to a spatial redox gradient (see above) there is a time-dependent modulation of cytosolic glutathione that correlates with neuronal differentiation.

### The GSH:GSSG ratio changes in differentiating SH-SY5Y cells

**Figure 4:**
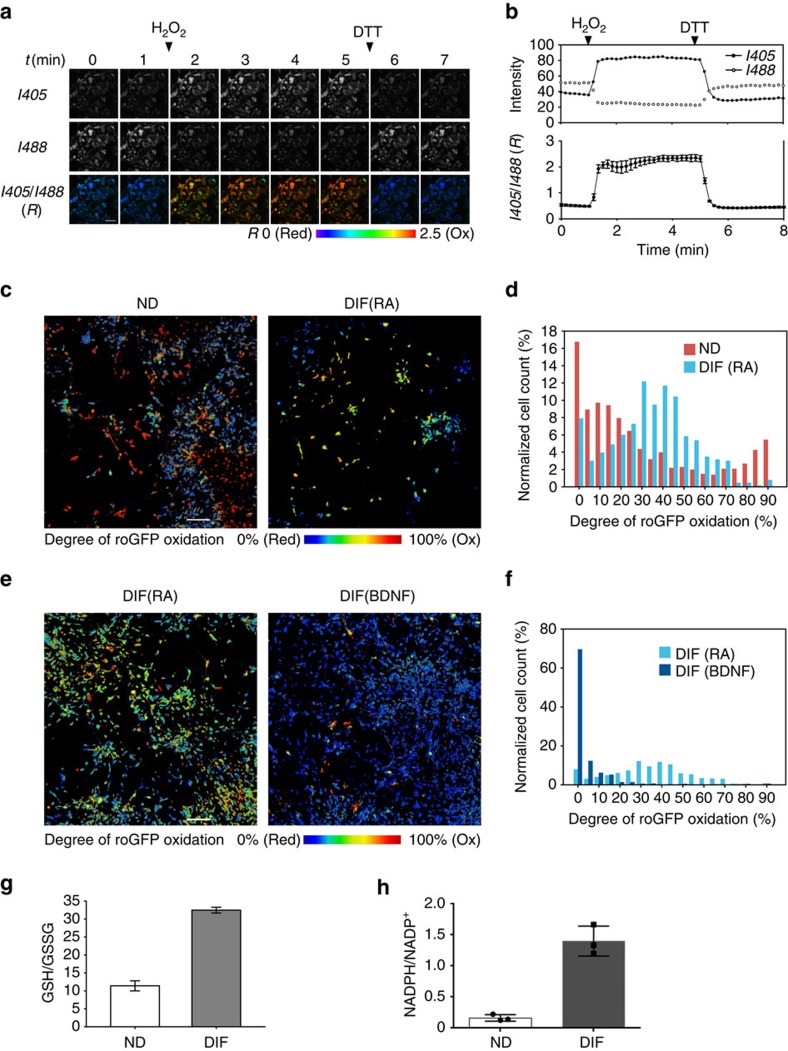
Cytosolic glutathione homoeostasis in SH-SY5Y cells changes during cell differentiation. (**a**) Grx1-roGFP2 was expressed in differentiated SH-SY5Y cells using adenovirus and its sensitivity to redox changes was examined. Cells were sequentially treated with 500 μM H_2_O_2_ and 2 mM DTT. Time-lapse images with 1 min interval are shown. Scale bars, 50 μm. (**b**) Top: changes in fluorescence intensity after illumination at *I488* and *I405* are plotted for one representative cell. Bottom: plot of average *R* value for 15 cells. (**c**,**e**) The analysis of cell population demonstrated large variation in redox states of non-differentiated SH-SY5Y cells and a significant change upon RA and BDNF treatments. (**d**,**f**) Histograms for images shown in **c**,**e**. To increase the sample size, another 1,125 μm × 1,125 μm area was scanned and analysed together (Supplementary Figs 12–14). *n*=1,079 cells (ND), 633 cells (RA) and 1,584 cells (BDNF). Scale bar, 200 μm. (**g**) Neuronal differentiation is associated with increase of GSH/GSSG ratio. The amounts of GSH and GSSG were measured spectrophotometrically using enzymatic assay; mean values are 11.4 and 32.5 for non-differentiated and differentiated cells, respectively (*n*=3). Each value is presented as mean±s.d. (**h**) The NADPH/NADP^+^ ratio increases 8.6-fold from 0.16±0.03 in non-differentiated SH-SY5Y cells to 1.40±0.14 in differentiated cells (*n*=3; Student's *t*-test, *P*<0.001). Squares and circles indicate each data point and bars represent s.d. The change in NADPH/NADP^+^ ratio is due to a decrease in NADP^+^ and an increase in NADPH levels (Supplementary Fig. 15a).

To independently evaluate the redox status of cytosolic glutathione during differentiation, we used SH-SY5Y cells. As in tissues, the ability of Grx1-roGFP2 to respond to redox changes was first verified by sequential treatments with H_2_O_2_ and DTT. The H_2_O_2_ treatment caused rapid increase of the *R* value, indicative of sensor oxidation, which was decreased upon treatment with DTT (Fig. 4a,b).

We then assessed the oxidation state of glutathione in non-differentiated cells, RA-treated cells, and BDNF-treated cells. The redox status of HEK293 cells was analysed for comparison (Supplementary Figs 10 and 11). The degree of sensor oxidation (*OxD*) was calculated from the *R* values using Hanson's equation. Cytosol of HEK293 was highly reduced (median *OxD*=5.2%; Supplementary Fig. 11). In contrast, non-differentiated SH-SY5Y cells showed a broad range of redox states, which segregated into the reduced (<35% *OxD*) and oxidized (>80% *OxD*) populations (Fig. 4c,d). Cells treated with RA yielded a more uniform redox distribution with a median *OxD* value of 40%, which falls in an intermediate range between the reduced and oxidized subpopulations found in non-differentiated cells. Upon full differentiation following BDNF treatment, glutathione in the vast majority of cells shifted towards a highly reduced state (<20% *OxD*; Fig. 4e,f). As a control, we analysed differentiated cells placed in a serum-containing medium for 1 day and confirmed that changes in the redox pattern induced by BDNF treatment is a serum-independent phenomenon. The GSH/GSSG ratio in non-differentiated and differentiated cells was also compared using an enzymatic assay. Neuronal differentiation led to an increase of the GSH/GSSG ratio (Fig. 4g), consistent with a shift towards a more reduced state of glutathione pair observed in the roGFP experiments. Altogether, our data indicate that cytosolic glutathione becomes more reduced upon neuronal differentiation.

Larger fraction of reduced glutathione implies an increased availability of nicotinamide adenine dinucleotide phosphate (NADPH), the main cellular reductant maintaining the redox status of GSH. To test this prediction we measured levels of NADPH in non-differentiated and differentiated SHSY-5Y cells and also calculated the NADPH/NADP^+^ ratio. Both were significantly higher in differentiated cells (Fig. 4h and Supplementary Fig. 15a) pointing to a potential mechanism through which differentiated cells maintain a larger fraction of reduced glutathione.

### Atox1 is more strongly reduced in differentiated cells

**Figure 5:**
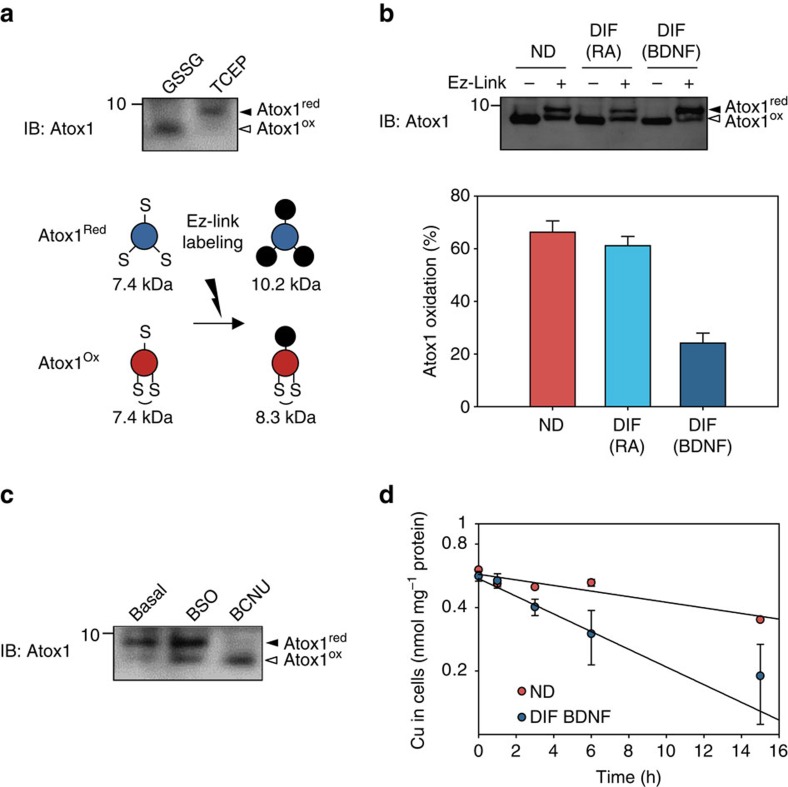
The redox status and copper-binding ability of Atox1 are regulated by cytosolic glutathione and change during SH-SY5Y cell differentiation. (**a**) The approach for evaluation of the redox status of endogenous Atox1. The three cysteines (indicated by ‘S') present in Atox1 (protein mass=7.4 kDa) are labelled with Ez-Link (indicated by black circles, mass=922 Da). The stoichiometry of labelling depends on the redox status. The upper panel shows the result of a control experiment where cell lysates were pretreated with either 1 mM reducing reagent tris(2-carboxyethyl)phosphine or 1 mM GSSG for 30 min. Labelled proteins were separated on 15% Tricine gel. The higher and lower bands represent reduced and oxidized forms of Atox1, respectively. (**b**) Oxidation state of Atox1 changes with neuronal differentiation. Percentage of oxidized Atox1 was estimated by densitometry of bands. Three independent blots (Supplementary Fig. 16) were analysed and each value is presented as mean±s.d. in the lower panel (*n*=3). (**c**) The redox status of Atox1 is regulated by the redox balance of cytosolic glutathione. Differentiated cells (DIF BDNF) were treated with either 0.5 mM buthioninesulfoxide or 0.1 mM BCNU for 6 h, where indicated. BCNU induces oxidation of cellular glutathione and, in the BCNU-treated cells, most of Atox1 was oxidized. (**d**) DIF BDNF showed higher copper efflux compared with non-differentiated cells. After treatment with 50 μM copper for 24 h, cells were placed in the basal medium and cellular copper amounts were measured at different time points by atomic absorption. Each value is presented as mean±s.d. normalized to protein amount (*n*=3). Plots were fit to single exponential decays with the rate constants of 0.080 h^−1^ (DIF BDNF) and 0.032 h^−1^ (ND).

To determine whether changes in the oxidation state of cytosolic glutathione affect the ability of Atox1 to bind copper, we examined the oxidation state of Atox1 copper-binding Cys-Gly-Gly-Cys site using a previously developed Cys-labelling method^10^. Reduced Atox1 has three free thiols Cys12 and Cys15 in the copper-binding site and Cys41 in a distant position (Fig. 5a, lower panel), whereas the oxidized Atox1 has only one (Cys41). Labelling free thiols with the Ez-Link maleimide-PEG11-biotin (MW 922) allows to distinguish the reduced and oxidized forms of Atox1 by difference in the protein mass (10.2 and 8.3 kDa, respectively; Fig. 5a upper panel). In non-differentiated cells, 66.2±4.3% of Atox1 was oxidized (Fig. 5b and Supplementary Fig. 16a–c). Differentiation decreased Atox1 oxidation (24.1±3.8%), in agreement with a more reduced state of cytosolic glutathione. We then treated differentiated cells with buthyl-*c*-nitrosourea (BCNU) to artificially induce glutathione oxidation. This treatment increased fraction of oxidized Atox1 from 19 to 79% (Fig. 5c), establishing that the redox status of Atox1 is regulated by cytosolic glutathione. Atox1 redox status was not affected by lowering glutathione level using buthioninesulphoximine, an inhibitor of γ-glutamylcysteine synthase, while combination of buthioninesulphoximine and hydrogen peroxide resulted in significant oxidation (Supplementary Fig. 16e). These results suggest that the oxidation state of Atox1 depends on the redox balance rather than the total amount of glutathione in the cytosol.

### Copper flux via the secretory pathway is redox-regulated

Atox1 functions as a cytosolic copper shuttle for the secretory pathway. Copper transfer to this compartment serves two functions: it enables functional maturation of copper-dependent enzymes, such as PAM and DBH, and removes excess copper from the cytosol via vesicle-mediated secretion. Oxidation of Atox1 metal-binding site is expected to decrease copper delivery to secretory vesicles, whereas reduction would facilitate this process. To test this prediction, we compared copper efflux from differentiated and non-differentiated cells as a measure of copper sorting to the secretory pathway. Cells were first loaded with copper, then transferred into a basal medium and loss of copper from cells was monitored with time (Fig. 5d). The retention of copper in both non-differentiated and differentiated cells followed a single exponential decay but had very different rate constants: 0.080 h^−1^ for differentiated cells and 0.032 h^−1^ for non-differentiated cells. The rate of copper efflux from differentiated cells was also determined in the presence of 10% fetal bovine serum (FBS); the rate was 0.076 h^−1^, again higher than in non-differentiated state. Thus, copper export from cells increases about 2.5-fold upon differentiation.

To determine whether glutathione oxidation would affect the rate of copper secretion, cells were treated with BCNU before measuring copper efflux. However, combination of BCNU and saturating levels of copper (necessary for the efflux assay) produced severe cytotoxicity. Consequently, we assessed effect of oxidation on copper delivery to the secretory pathway under regular growth conditions by measuring secretion of DBH before and after BCNU treatment. Soluble copper-bound form of DBH is secreted and can be measured in the extracellular medium^28^. BCNU treatment decreased the amount of secreted DBH; reciprocally more unprocessed form of DBH was observed inside the cell (Supplementary Fig. 17). These effects were not due to lower expression/abundance of ATP7A or Atox1, which remain unchanged (Supplementary Fig. 18). Thus BCNU treatment and associated glutathione oxidation decreases copper delivery to the secretory pathway.

## Discussion

**Figure 6:**
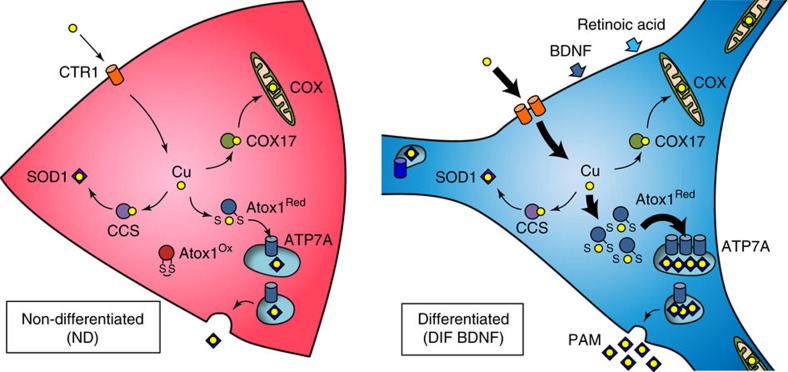
Redox-regulated remodelling of cellular copper fluxes during neural differentiation of SH-SY5Y cells. The intracellular routes of copper (yellow circle) are indicated by arrows. Bold arrows represent the pathways upregulated upon differentiation.

Normal function of central nervous system requires proper copper homoeostasis. In neuronal cells, copper is utilized for neurochemical activities as well as general metabolism. Our results indicate that neuronal cells modulate their copper metabolism upon differentiation in conjunction with changes in redox homoeostasis. We demonstrate that the demand for copper in the secretory pathway increases with neuronal differentiation and that metal compartmentalization is facilitated by changes in the transcriptome and cellular redox state (Fig. 6). Our measurements of glutathione balance in developing motor neurons *in vivo* demonstrate that redox state of cytosolic glutathione correlates with the stage of neuronal differentiation. In addition to the normal physiological processes, such as differentiation, glutathione redox balance can also be altered in disease or as a result of aging. Our results suggest that changes in glutathione oxidation can be translated and amplified (through the activities of copper-dependent enzymes) into specific metabolic responses.

Copper-dependent enzymes function in different intracellular compartments (cytosol, mitochondria and the lumen of secretory vesicles) and their functional maturation requires targeted delivery of copper to the corresponding compartments. In the secretory pathway, lysyl oxidases (LOX1-5), ferroxidases (ceruloplasmin^29^), SOD3 (ref. 30) and various monooxygenases (such as DBH^31^ and PAM^32^) depend on timely delivery of copper by ATP7A or ATP7B with the help of the chaperone Atox1 (ref. 33). Our data indicate that some of these enzymes, such as PAM and DBH, are expressed at higher levels in differentiated neurons and the increased protein production coincides with upregulation of ATP7A and Atox1 expression (at the mRNA and protein levels) to meet higher demand for copper in the secretory pathway. Similar co-regulation between PAM and ATP7A was reported for amygdala interneurons^34^. We also found that ATP7A levels in differentiating cells are increased in response to RA but not BDNF, consistent with the previous finding that ATP7A is a target gene for RA receptor β-2 (ref. 35). In addition to upregulation of ATP7A, we observed increased expression of other genes involved in copper metabolism: in mitochondria (COX17 and COX1) and copper uptake transporters (CTR1 and CTR2). This observation suggests that cell differentiation involves significant remodelling of the intracellular copper-trafficking system.

Differentiated cells showed higher copper efflux (Fig. 5d) and yet higher copper content (Fig. 1f). This observation suggests that the rate of copper uptake mediated by CTR1 (which is upregulated in differentiated cells) exceeds the rate of copper secretion mediated through vesicle fusion, producing on a balance, higher steady-state levels of cellular copper. In addition, some upregulated copper-binding proteins such as PAM remain in a cell, thus contributing to a higher copper content in differentiated cells. Altogether these data suggest a model where neuronal differentiation is associated with simultaneous activation of copper absorption and secretion, generating higher copper flow through the secretory pathway to supply copper for maturation of copper-dependent enzymes. Although copper transport to the secretory pathway may not be obligatory for neuronal differentiation, the enhanced copper delivery to secretory pathway is required for normal neurochemical activities of neurons, as demonstrated by the reduced DBH secretion in the BCNU-treated cells. Human patients with Menkes disease provide a real-life example of the essentiality of copper delivery to the secretory pathway for normal neuronal function. These patients have insufficient function of ATP7A resulting in abnormal neuronal arborization and myelination, pathologic electrical activity and epilepsy as early as months after birth^36^.

Cellular redox environment is controlled by multiple components including glutathione, cysteine, NADPH, thioredoxin and other redox-active molecules. These redox nodes are chemically and spatially independent from one another^37^. Previous data have suggested that the production of ROS increases with differentiation in hematopoietic cells^38^ and colonic epithelial cells^39^. These observations may appear inconsistent with our findings showing increased reduction of glutathione. However, higher levels of ROS may not necessarily increase oxidation of glutathione, because the redox status of cytosolic glutathione pair depends on many factors (the rate of production and subcellular location of ROS, SOD1 activity, glutathione peroxidase activity, glutathione synthesis, glutathione transport and other factors). Physiologic oxidants such as H_2_O_2_ act as potent stimulators of cellular energy production^40^, and it is possible that increased ROS levels may be balanced by enhanced reduction of NADPH/NADP^+^ pair and/or glutathione pair. Such relationship is seen in cancer cells, which have high ROS production along with high-antioxidant capacity^41^. Similarly, neurons transiently produce nitric oxide^42^ and maintenance of its antioxidant capacity depends on pentose-phosphate pathway, where glucose is metabolized to generate NADPH for converting oxidized glutathione to the reduced state. Supporting this possibility, NADPH level as well as NADPH/NADP^+^ ratio are indeed significantly elevated upon differentiation in SH-SY5Y cells (Fig. 4h).

Quantitative analysis of individual redox nodes in animal models has been technically challenging, especially because the redox status of subcellular compartments such as ER, mitochondoria and cytosol differs significantly. In the milestone work by Gutsher *et al*.^26^, Grx1-roGFP2 was designed to specifically report status of glutathione pair within specific compartments and probe real-time redox changes during starvation, mitochondrial depolarization and immune response in cultured mammalian cells. We demonstrate utility of cytosolic Grx1-roGFP2 for studies in neuronal cells in the developing spinal cord. Our experiments identified variations in the redox status of cytosolic glutathione that appear to correlate with neuronal differentiation. Furthermore, motor neurons at the later HH stage 23 showed more reduced cytosolic environment compared with cells at the HH stage 20 embryos. In cultured cells, we observed analogous variation in the redox status of proliferating cells and a uniform glutathione reduction following BDNF treatment. Previous studies in other cell types demonstrated correlation between the glutathione level and cell cycle progression^43^,^44^. In CHO cells, progression from the G1 to G2/M phase is associated with an increase in the cellular GSH level, potentially providing optimal redox environment for oxidation-sensitive proteins activated in the G2/M phase^44^. Similar conclusion was made for A549 lung carcinoma cell line^43^. We hypothesize that the observed redox variability in proliferating cells (neuroprogenitors) reflects the cell cycle phase-dependent oscillations. Neuroblastoma and neuroprogenitors are actively cycling cells, while differentiated neurons are quiescent (arrested in G0) perhaps explaining why non-differentiated cells showed higher degree of cell-to-cell redox variability. This difference in cellular GSH:GSSG ratio may underlie a preferential cytotoxic effect of the redox modulator BCNU towards rapidly growing glioblastomas^45^. Better understanding of cellular redox nodes in normal growth and differentiation may yield novel approaches to control cell cycle progression.

## Methods

### Plasmid constructs and adenovirus vectors

Oligonucleotides used in the study are listed in Supplementary Fig. 17a. Original construct of Grx1-roGFP2 (pEIGW/Grx1-roGFP2) was kindly provided by Dr Tobias Dick (German Cancer Research Center)^26^. Grx1-roGFP2 cDNA was transferred to a multi-cloning site in pCDNA5 vector (Invitrogen) using SmaI/NotI sites in the original construct and *Eco*RV/*Not*I sites in the pCDNA5 vector, resulting in pCDNA5/Grx1-roGFP2. To develop adenoviral construct, Grx1-roGFP2 cDNA was transferred to multi-cloning site in pShuttle-CMV (Addgene) using BamHI/NotI sites in pCDNA5/Grx1-roGFP2 and BglII/NotI sites in pShuttle-CMV vector, resulting in pShuttle-CMV/Grx1-roGFP2. This construct was linearized by *PmeI* digestion and transformed into BJ5183 bacteria carrying pAdEasy-1 for homologous recombination between these two plasmids. Colonies were selected by kanamycin and screened for the presence of AdEasy plasmid carrying Grx1-roGFP2 expression cassette. The resulting construct (pAdEasy-CMV/Grx1-roGFP2) was linearlized by *Pac*I digestion and transfected into HEK293A cells. Virus packaging was confirmed by presence of virus plaques. Virus was amplified by three cycles of re-infection. Obtained virus suspension (AdGrx1-roGFP2) was stored at 4 °C for short-term storage (typically within 2 weeks) and −80 °C for long-term storage (up to 6 months).

To generate GFP-fused Atox1, Atox1 cDNA was amplified by PCR using oligonocleotides Atox1_f_NheI and Atox1_r_EcoRI and pCDNA3/Atox1-FLAG^10^ as a template. The resulting DNA fragment was ligated into pCDNA3.1/EGFP (will be described elsewhere) using NheI/EcoRI sites to position Atox1 cDNA in frame to the 5′ side of the EGFP-coding sequence. For the information about the plasmid constructs used for absolute quantitation of mRNA, see the section ‘quantitative real-time PCR'.

### Cell lines and culture conditions

HEK293 cell line was kindly provided by Dr Hubbard, Johns Hopkins University. SH-SY5Y cell line was obtained from ATCC. HEK293A was maintained in 1:1 mixture of minimum Eagle's medium (MEM) and F-12 Ham's medium supplemented with 10% FBS (v/v). Neuronal differentiation of SH-SY5Y was induced by sequential treatments with 10 nM RA for 5 days and 50 ng ml^−1^ BDNF in serum-free medium for subsequent 3 days^16^.

### Production of antibodies

Rabbit anti-Atox1 antiserum was generated by SDIX (Newark, Delaware) using a purified recombinant Atox1 as an antigen. Purified untagged Atox1 was prepared using previously described procedure and construct^10^. Briefly, intein-Atox1 fusion protein was expressed in *E. coli* strain BL21(DE3) transformed with the pTYB12/Atox1 plasmid^10^ and purified from a soluble cell fraction using a chitin resin (New England Biolabs, S6651L). Purified Atox1 was eluted following the DTT-induced cleavage of intein, dialyzed against phosphate-buffered saline (PBS)-NaCl (50 mM sodium phosphate, pH 7, 150 mM NaCl), and concentrated using an Amicon ultrafiltration unit (EMD Millipore, UFC900308). Protein purity was assessed by 15% Tricine SDS-PAGE^46^ and protein concentration was determined by Bradford assay (Bio-Rad, 5000205) using BSA as a standard. Two rabbits were immunized by injecting the purified Atox1.

### Immunoblot analysis

Cells were typically cultured in a six-well plate or 100-mm dish, mechanically collected without trypsin treatment, washed with PBS twice, and resuspended in PBS supplemented with Complete Protease Inhibitor Cocktail (Roche). Cells were lysed by homogenization and debris as well as nuclei were removed by centrifugation at 5,000 r.p.m. for 20 min. Protein concentration of the resulting cleared lysate was determined by BCA assay. Before electrophoretic separation of proteins, each sample was combined with an equal volume of 2 × Laemmlli sample containing 50 mM DTT. Proteins were then resolved on either 15% Tricine SDS-PAGE^46^ (Atox1) or 8% Laemmlli SDS-PAGE^47^ and transferred to PVDF membrane at 100 V for 120 min in towbin buffer. Primary antibodies used in immunoblotting were: mouse monoclonal anti-FLAG M2 clone (Sigma, F1804), mouse monoclonal anti-Atox1 (Sigma, wh0000475m1), rabbit anti-Atox1 antiserum (described in the previous section), mouse monoclonal anti-ATP7A (Santa Cruz, 376467), rat polyclonal anti-ATP7B (ref. 3), mouse monoclonal anti-tubulin (Sigma, T8203), mouse monoclonal anti-microtubule-associated protein 2 (Sigma, M4403), and mouse monoclonal anti-actin (Sigma, A5441). Secondary antibodies were: goat polyclonal anti-mouse IgG HRP-conjugate (Santa Cruz, SC-2005), goat polyclonal anti-rabbit IgG HRP-conjugate (Santa Cruz, SC-2004). All antibodies were used at a dilution of 1:1,000.

### Immunostaining of cultured cells

Immunostaining was performed as previously described^9^. Cells were cultured on collagen-coated cover slips in a 12-well plate, fixed with 3.7% paraformaldehyde in PBS for 20 min, permealized with 0.2% (w/v) Triton X-100 in PBS for 10 min, and blocked with 1% (w/v) BSA/1% (w/v) gelatin in PBS. Primary antibodies used in cell staining were mouse monoclonal anti-FLAG M2 clone (Sigma, F1804), rabbit polyclonal anti-ATP7A (Hycult Biotech, HP8040), mouse monoclonal anti-ATP7A (Santa Cruz, 376467), mouse monoclonal anti-EEA1 (BD Biosciences, 610456), mouse monoclonal anti-Rab11 (BD Biosciences, 610656), mouse monoclonal anti-Lamp1 (developmental studies hybridoma bank, H4A3-s), and sheep monoclonal anti-TGN46 (Gene Tex, GTX74290). Secondary antibodies used for cell staining were: goat polyclonal anti-mouse IgG Alexa488-conjugate (Molecular Probes, A-11001), goat polyclonal anti-rabbit IgG Alexa555-conjugate (Molecular Probes, A-21428). Cells were imaged with a Zeiss LSM 710. Images were processed with ZEN and Image J. Anti-EEA1, anti-Rab11, and anti-Lamp1 antibodies were kindly provided by Dr Rajini Rao (Johns Hopkins University). All antibodies were used at a dilution of 1:200.

### Analysis of copper levels by atomic absorption

Cells were washed, collected, suspended in ultrapure water and immediately mixed with an equal volume of 2% SDS. Lysate was incubated at 90 °C for 15 min. Protein concentration was determined by bicinchoninic acid assay. Typically ∼20 μl of lysate containing 100 μg protein was combined with 20 μl of 40% nitric acid in a glass test tube and incubated at 50 °C for 20 min. The sample was combined with 360 μl of ultrapure water and debris was removed by centrifugation at 5,000 r.p.m. for 5 min. Copper concentration in the supernatant was measured using atomic absorption spectroscopy (AA-6650G, Shimadzu, Columbia, MO), and a copper/protein molar ratio was averaged for each sample in three independent experiments. For copper analysis of microsomes, cells were suspended in PBS and homogenized. Debris and nuclei were removed by centrifugation at 5,000 r.p.m. for 15 min. Microsomes were collected by centrifugation at 20,000*g* for 30 min. Pelleted microsomes were resuspended in 50 μl of ultrapure water and combined with an equal volume of 2% SDS, followed by the same procedure described above for cell lysates.

### Quantitative real-time PCR

mRNA levels in non-differentiated, RA-treated and BDNF-treated cells were analysed by quantitative real-time PCR (qPCR). Three biological replicates were prepared for each condition (*n*=3). Total RNA was isolated from cells with RNeasy kit (Qiagen) and corresponding cDNA pools were synthesized using Fast-Strand cDNA synthesis kit (Roche). RT–PCR was performed with SYBR green (Applied Biosystems) on an ABI 7500 Sequence Detection System (Applied Biosystems). For ddCt analysis, GAPDH levels were used for normalization. Primers used in this study are listed in Supplementary Fig. 19a. For absolute quantitation, plasmid standards were prepared by inserting each amplicon into pCR2.1-TOPO vector. DNA concentrations of plasmid standards were determined by absorbance at 260 nm. The standard curves are shown in Supplementary Fig. 19b.

### Redox status of cytosolic glutathione

The redox status of glutathione pair was determined using ratiometric fluorescence sensor, Grx1-roGFP2 (ref. 26). Cells were cultured on collagenized 35 mm Fluoro Dishes. Grx1-roGFP2 cDNA was delivered into cells by transfecting 2 μg of pCDNA5/Grx1-roGFP2 using Lipofectamin 2000 (HEK293A) or infecting 200 μl of AdGrx1-roGFP2 viral suspension (SH-SY5Y). Two days after transfection/infection, culture medium was changed to 1 ml of roGFP assay medium: 1 ml of phenol-red free DMEM supplemented with 10% (v/v) FBS and 50 mM HEPES, pH 7.4. For maximum (100%) degree of sensor oxidation (*OxD*), one of the HEK293A samples was treated with 0.2–0.5 mM H_2_O_2_ for 2 min, fixed with 3.7% paraformaldehyde, washed with PBS twice, placed into 1 ml of roGFP assay medium. In some experiments, live cells were also used as control. Samples were imaged with a Zeiss LSM 710 equipped with blue diode and Ar/He lasers. Each image was taken for two tracks (T1 and T2) with distinct excitation (488 and 405 nm, respectively) and a single emission range 540–560 nm. The ratio of intensities for T2 and T1 (*I*_405_/*I*_488_) represents a measure of oxidation of Grx1-roGFP2. For maximum reduction of sensor 1/10 volume of 20–50 mM DTT was added. This treatment produces immediate and complete reduction of Grx1-roGFP2, which is taken as 0% sensor oxidation. Fluorescence ratio *I*_405_/*I*_488_ was then converted to *OxD*, using Hanson's equation. To exclude the possibility that the observed difference in glutathione redox status was due to different cell densities, we analysed cells at various time points. In non-differentiated cells, *OxD* value was constantly higher than 60% even at day 2 when cell density was lower than differentiated state (BDNF-treated cells at day 7 and 8; Supplementary Fig. 15b).

### Determination of GSH/GSSG ratio and NADPH/NADP^+^ ratio

Cells were cultured on 150-mm dish, washed with PBS and resuspended in lysis buffer (50 mM Tris-HCl pH 8.0, 1 mM ethylenediaminetetraacetic acid). Cells were lysed by flash sonication and centrifuged at 20,000*g* for 15 min to remove the debris. Parallel samples (200 μl each) were prepared for determining amounts of reduced glutathione (*GSH*_red_) and total glutathione (*GSH*_total_). To measure (*GSH*_red_), 0.25 mM DTNB was added and absorbance at 412 nm (*A*_412_) was recorded. Glutathione solution (0, 10, 20, 50, 100 and 200 μM dissolved in lysis buffer) was used as standard. To measure (*GSH*_total_), samples were pretreated with 0.6 IU GSH reductase and 0.25 mM NADPH, followed by DTNB reaction and A_412_ measurement. The relative amount of oxidized glutathione was then calculated by (*GSH*_total_)−(*GSH*_red_). The total protein concentration was measured by BCA Protein Assay Kit (Pierce).

NADP^+^ and NADPH levels were measured, separately, using EnzyChrom NADP^+^/NADPH Assay kit (BioAssay Systems, ECNP-100) in non-differentiated and differentiated SH-SY5Y cells (10^5^ cells per sample). The absorbance at 565 nm (OD_565_) was monitored after 30 min incubation and values were converted to NADP(H) concentrations using a standard curve.

### Electroporation of roGFP into chicken spinal cord

For Grx1-roGFP2 *in vivo* experiment, chicken embryos at HH development stage (HH stage) 12–14 were electroporated unilaterally with 4–6 μg μl^−1^ Grx1-roGFP2 construct and analysed at HH stage 20-21 (ref. 25). Embryos were dissected in cold PBS containing 20 mM N-ethylmaleimide (NEM) to block oxidation and maintained in the same solution for 30 min at 4 °C. NEM was removed by rinsing embryos once with PBS followed by 4% paraformaldehyde fixation for 90 min at 4 °C. Embryos were washed with PBS and equilibrated in 30% sucrose before embedding and sectioning. Control embryos were treated with 20 mM H_2_O_2_ or 20 mM DTT at room temperature for 10 min before blocking with NEM. Effective preservation of the redox status of roGFP by NEM was verified in HEK293 cells (Supplementary Fig. 6). Cryostat sections (12.5 μm) were imaged directly for Grx1-roGFP2 or immunostained to detect neuronal progenitors (Sox2, Santa Cruz, 2c-17320), postmitotic neurons (p27Kip1, BD Biosciences, 610241), motor neuron progenitors (Olig2, kindly provided by Dr B. Novitch) and postmitotic motor neurons (Isl1/2, kindly provided by Dr T.M. Jessell) as previously described^25^,^48^. Cy3- or Cy5-conjugated secondary antibodies were from Jackson Immunoresearch (715-605-150; 711-605-152, 705-165-147, 706-165-148). Anti-PAM antibody was kindly provided by Dr Betty Eipper.

### The redox status of Atox1 in cells

The redox status of endogenous Atox1 in SH-SY5Y cells was determined using protocol previously described for studies of the recombinant Atox1 in HEK293 cells^10^. SH-SY5Y cells were cultured on a 6-well or 100-mm dish, mechanically collected and washed with PBS. The cells were resuspended in 250 μl of 10 mM MOPS, pH 7.0. After 10 min of incubation on ice, cells were homogenized in a Dounce homogenizer. After adding 250 μl of buffer containing 50 mM MOPS, pH 7.0, 0.5 M sucrose, 0.3 M NaCl, the cells were again homogenized. The lysate was cleared by centrifugations at 20,000*g* for 30 min. Supernatant was recovered, soluble proteins were precipitated by adding 10% TCA, and pelleted by centrifugation at 500 g. After acetone wash and air-drying, the protein pellet was dissolved in 100 μl of Laemmli sample buffer containing 4 M urea, 0.5 mM bathocuproinedisulfonate, and 1 mM ethylenediaminetetraacetic acid. The proteins were labelled with 2 mM EZ-Link maleimide-PEG11-biotin (Pierce) at room temperature for 3 h. The reaction was quenched by addition of 1/10 volume of 500 mM cysteine. After adding 1/10 volume of 500 mM DTT, the labelled samples were resolved on 15% Tricine gel for immunoblotting (see the ‘Immunoblot analysis' section for details). Rabbit anti-Atox1 antibody was used because its immunoreactivity was not affected by modification of Cys in Atox1; epitope recognition by mouse monoclonal anti-Atox1 antibody was significantly blocked by Cys-labelling (Supplementary Fig. 16d). Incorporation of EZ-Link maleimide-PEG11-biotin into Atox1 was identified by an upward shift in electrophoretic mobility of labelled protein. Densitometric analysis was performed by Image J.

### Statistical analyses

All numerical data are presented as the mean±s.d. Pairwise comparisons between different groups were performed using Student's *t*-test (two-tailed, unpaired). A difference was considered to be statistically significant when *P* value was <0.05.

## Additional information

**How to cite this article:** Hatori, Y. *et al*. Neuronal differentiation is associated with a redox-regulated increase of copper flow to the secretory pathway. *Nat. Commun.* 7:10640 doi: 10.1038/ncomms10640 (2016).

## Supplementary Material

Supplementary Information Supplementary Figures 1-19
