# Supplementary material for: Neuronal differentiation is associated with a redox-regulated increase of copper flow to the secretory pathway
Source: Nat Commun. 2016 Feb 16;7:10640. doi: 10.1038/ncomms10640 (PMC4757759; doi:10.1038/ncomms10640)
Supplement: Supplementary Information — Supplementary Figures 1-19 [file ncomms10640-s1.pdf]

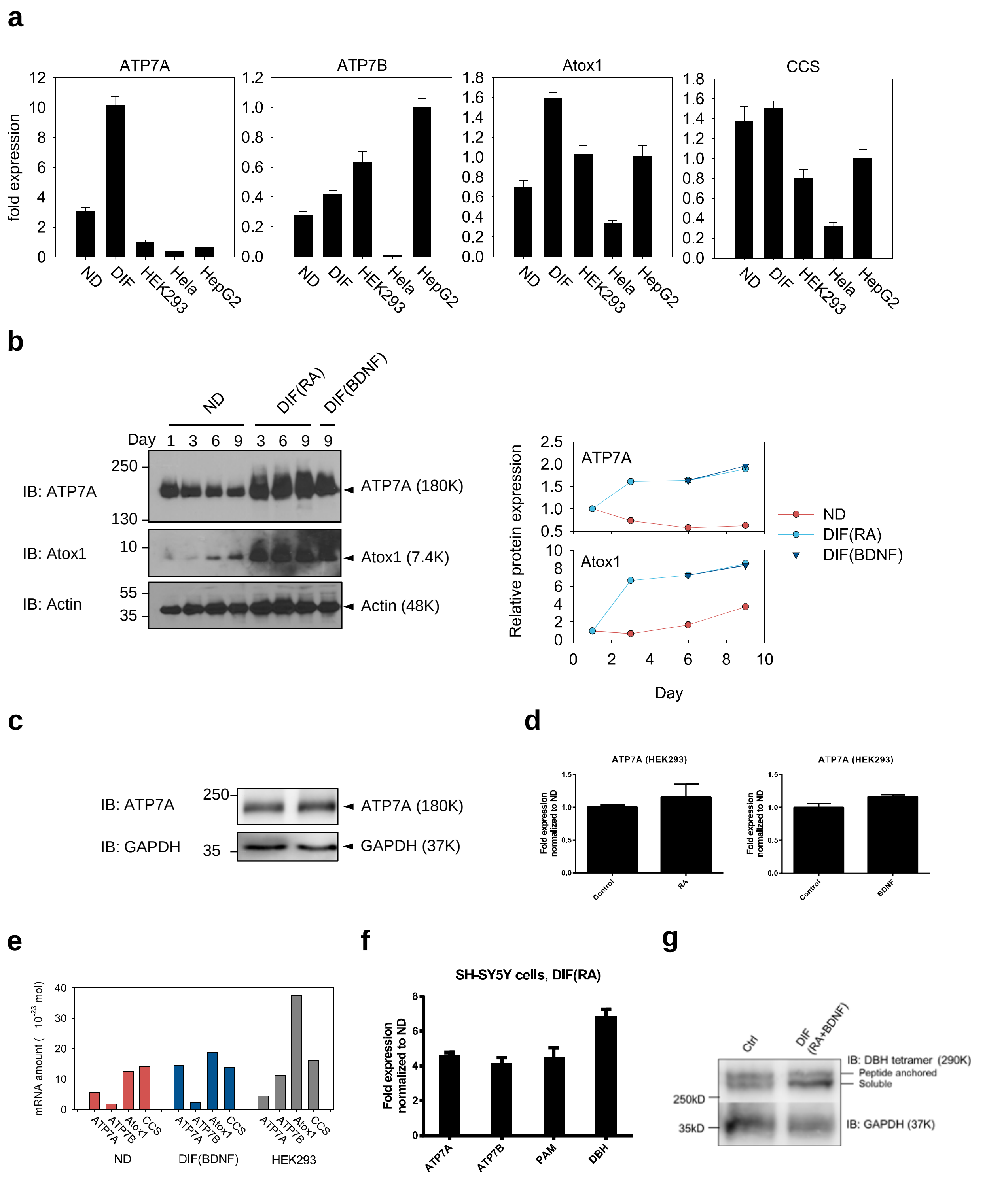

Supplementary Figure 1 | Gene expression profile in non-differentiated and differentiated SH-SY5Y cells. (**a**) Differentiation of SH-SY5Y cells is associated with upregulated expression of Atox1 and ATP7A. The mRNA levels of four representative copper-related genes were compared by ΔΔCt analysis for nondifferentiated and differentiated SH-SY5Y cells as well as other cell types (HEK293, HeLa, and HepG2). GAPDH was used as reference gene. Values

were normalized to HeLa (ATP7A), HepG2 (ATP7B, CCS), and HEK293 (Atox1). (**b**) Upregulation of ATP7A and Atox1 is induced by retinoic acid treatment. Time-dependent change of Atox1 and ATP7A expression was examined by western blotting; whole cell lysate was analyzed. Band intensity was quantified and normalized to the value for day 1. (**c**) Neither retinoic acid or BDNF treatment caused upregulation of ATP7A in HEK293 cells. HEK293 cells were used as non-differentiating cell culture model to test for ATP7A level upon retinoic acid (RA) treatment. ATP7A protein levels remained constant upon RA treatment. Equal protein amount of whole cell lysate (30 μg per lane) was loaded. (**d**) In HEK293 cells, neither retinoic acid (left) nor BDNF (right) did increase mRNA level of ATP7A. Values are normalized to non-differentiated cells. (**e**) At mRNA level, ATP7A is markedly abundant compared to ATP7B in SH-SY5Y. Absolute mRNA amounts of ATP7A, ATP7B, Atox1, and CCS were determined by real time qPCR. Standard curve for each gene is shown in Supplementary Fig. 19b. Equal amount of total RNA was analyzed. (**f**) Differentiation-dependent upregulations of ATP7A, ATP7B, PAM, and DBH were confirmed by using β-actin as reference gene. SH-SY5Y cells were treated with retinoic acid (see method in the main text) and mRNA levels of indicated genes were analyzed by qPCR. β-actin was used as reference gene. Values are normalized to non-differentiated cells. (**g**) DBH protein level increases upon complete differentiation of SH-SY5Y cells. Cells were differentiated by sequential treatments with retinoic acid (RA) and BDNF. Whole cell lysate was analyzed for DBH protein level by western blotting. Same amount of protein was loaded to each lane. Upregulation was judged by increase of the smaller band which represents processed soluble form of DBH. On the other hand, non-processed membrane-bound form (peptide anchored) remained largely unchanged.

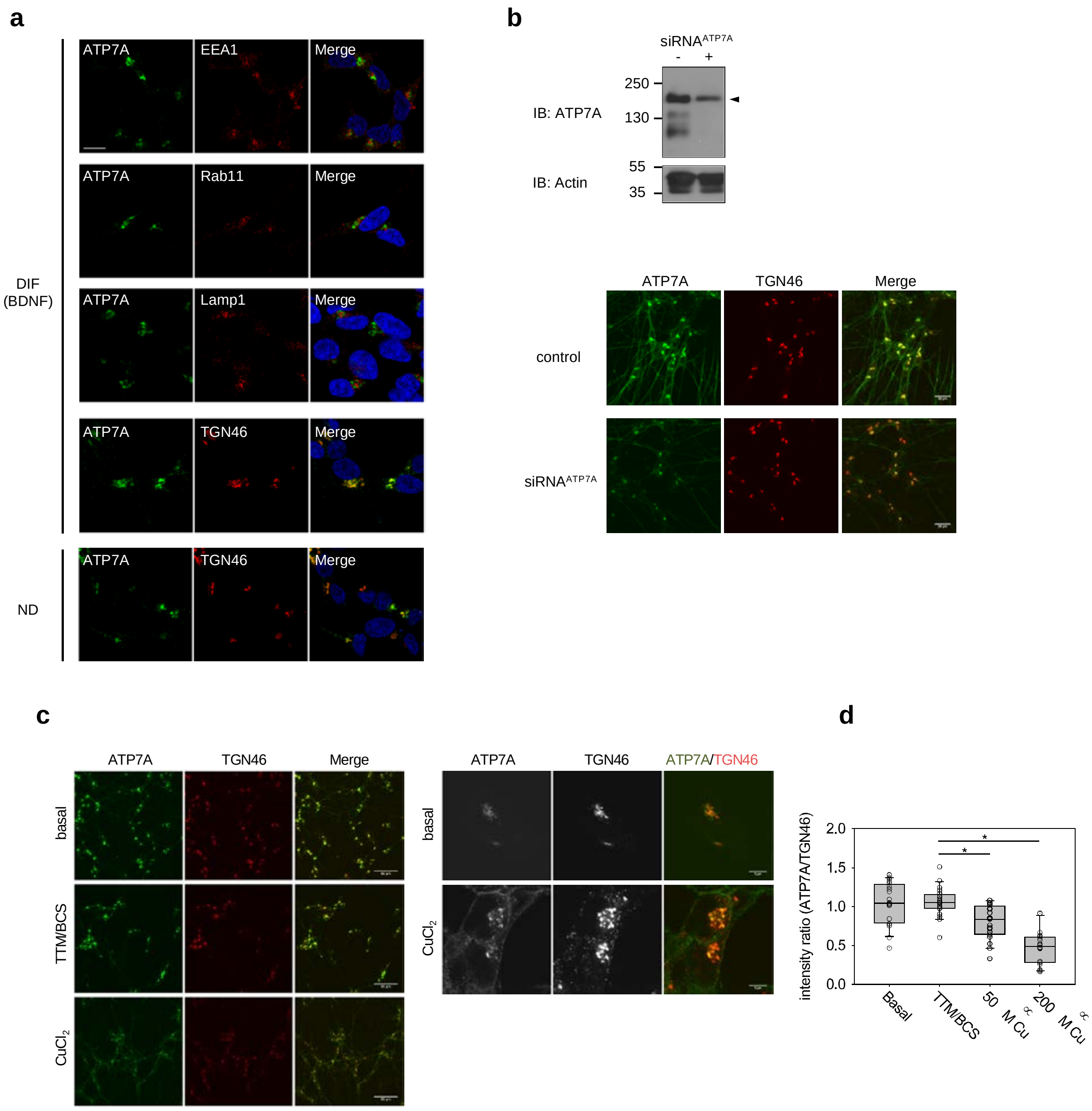

Supplementary Figure 2 | ATP7A is localized to TGN and vesicles independently of differentiated state of SH-SY5Y. (**a**) ATP7A is localized within TGN and vesicules that are distinct from the endosomal or lysosomal compartments. Differentiated SH-SY5Y cells were fixed with PFA and immunostained for TGN (TGN46), endosome (EEA1), recycling vesicle (Rab11), and lysosome (Lamp1) markers. Nucleus was stained with DAPI (blue). Similar pattern was observed for non-differentiated cells; representative colocalization with TGN is shown. Three different areas were imaged and colocalization of ATP7A and various markers were evaluated using Pearson's product-moment coefficients (main figure 1h). Three replicate samples were prepared and analyzed. Each value is presented as mean±SD (n=3). (**b**) Both perinuclear and vesicular staining represents ATP7A. Specificity of the observed signals was verified by treating cells with siRNA (25 nM, 3 days), where indicated. Images were taken for control and siRNA-treated samples using exactly the same microscope setting. Focal plane was adjusted so that TGN46 signal was maximized. Down-regulation of ATP7A was confirmed by

western blotting. (**c**) Effect of high copper treatment on ATP7A distribution pattern. Cells were treated with 200 μM $CuCl_2$ for 6 h or a combination of copper chelators: 20 μM tetrathiomolybdate (TTM) and 100 μM bathocuproinedisulfonate (BCS) for 24 h. Colocalization with TGN decreases upon copper treatment, which is consistent with previously demonstrated copper-dependent trafficking of ATP7A from TGN to vesicles. The same microscope setting was used for different samples. Focal plane was adjusted so that TGN46 signal was maximized. Scale bars, 50 μm and 5 μm in left and right panels. (**d**) Quantitative analysis on the images shown in *c* left. The result for 50 μM $CuCl_2$ treatment is also included. Signal intensities of ATP7A within TGN regions were measured and normalized to TGN intensities. This ratiometric analysis allows a semiquantitative estimation of ATP7A amount per unit area of TGN. *significant at $P<0.001$.

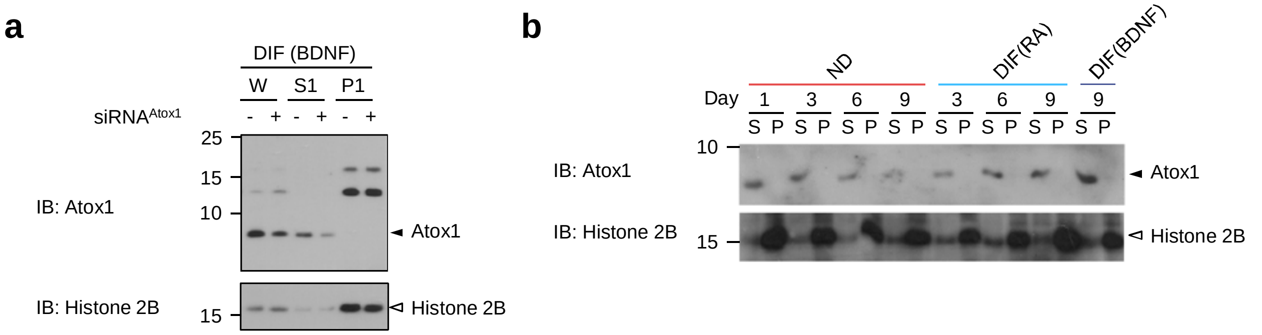

Supplementary Figure 3 | Atox1 is detected in the cytosol fraction. (**a**) Major pool of endogenous Atox1 is present in the cytosolic fraction. Cytosolic and nuclear fractions were prepared from differentiated SH-SY5Y cells and analyzed by western blotting. Histone 2B serves as a nuclear marker (open arrow head). To assess the specificity of anti-Atox1 antibody, cells were treated with siRNA (25 nM, 3 days) where indicated. Specific band of Atox1 is indicated by a closed arrow head; upper bands represent other proteins. (**b**) Cytosolic (S) and nuclear (P) fractions were prepared from SH-SY5Y cells at various stages of neuronal differentiation. Each fraction was analyzed for the presence of endogenous Atox1 by western blotting. Equal sample volume was loaded to each lane. Nuclear fraction is identified by presence of Histone 2B.

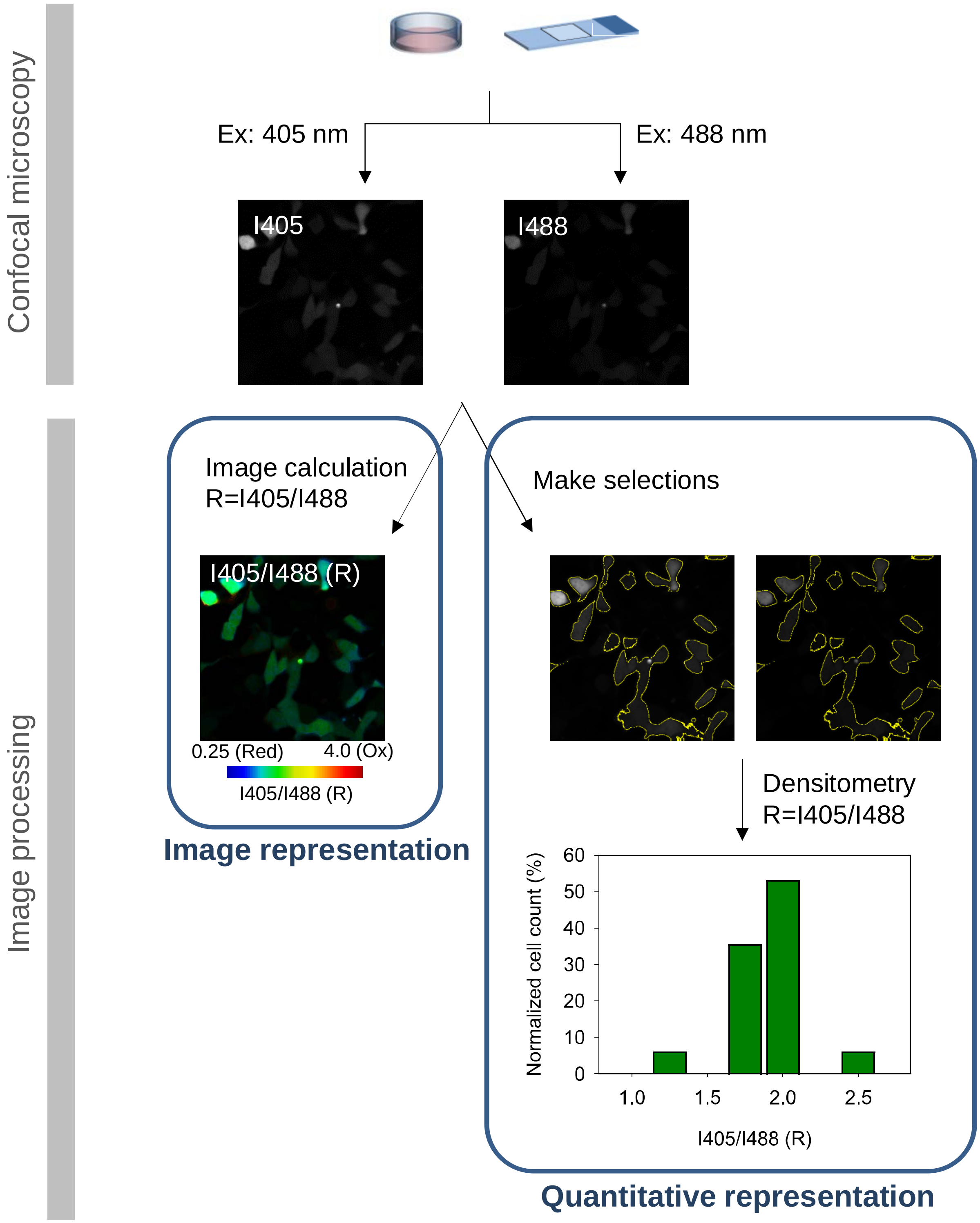

Supplementary Figure 4 | GRX1-roGFP analysis. Either live cells or fixed tissues expressing GRX1-roGFP were imaged following excitation at I405 and I488. For ratiometric image, the intensity values following excitation at I405 were divided by those at I488.

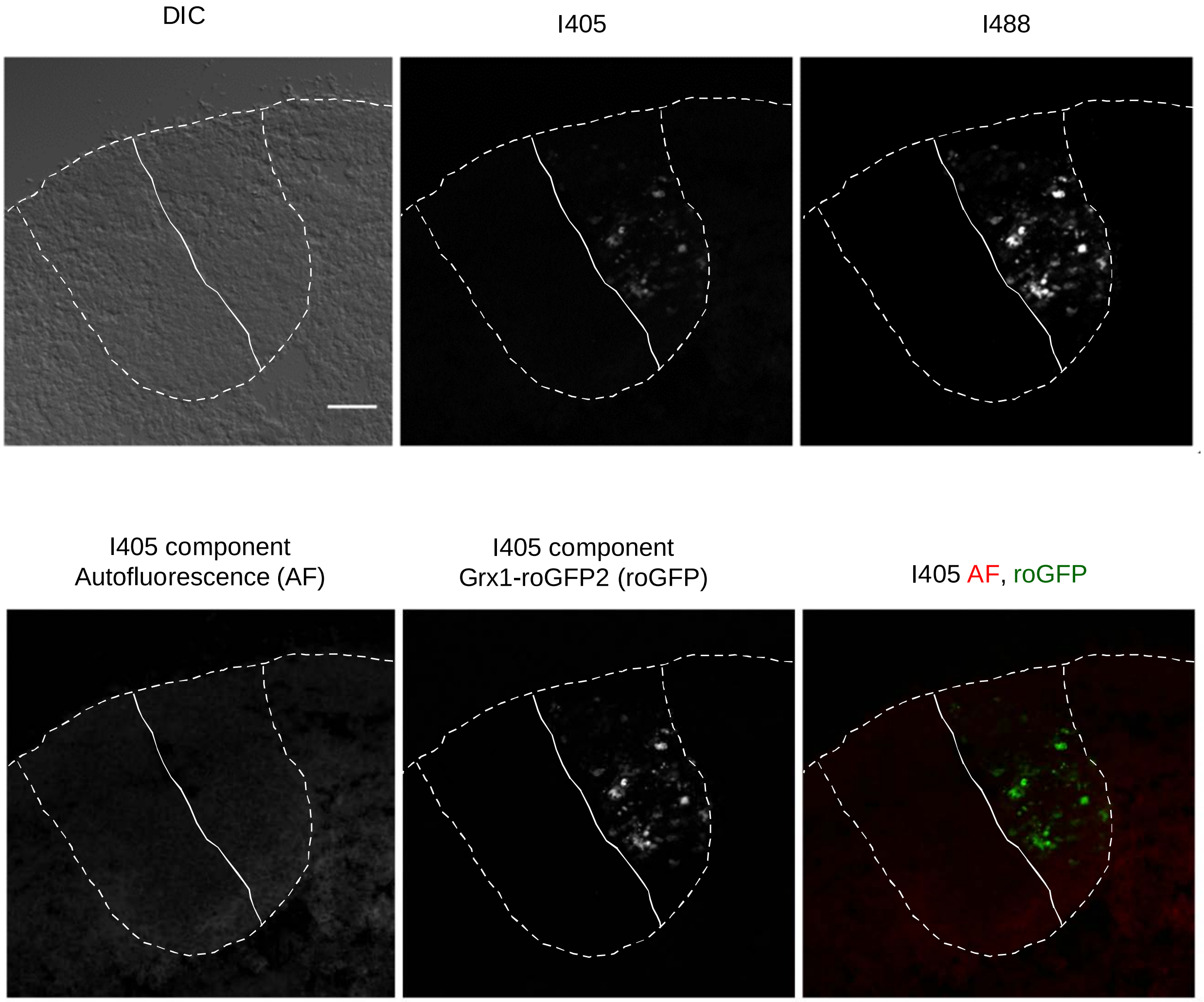

Supplementary Figure 5 | Preparation of confocal images for Grx1-roGFP analysis. The procedure of image processing for roGFP analysis is shown. Grx1-roGFP2 (roGFP) was expressed in chicken spinal cord. The boundaries of the spinal cord are marked by hatched white line. The midline is marked by a solid white line (change midline to solid line here) Six micrograms of pCDNA5/Grx1-roGFP2 plasmid were electroporated into spinal cord *in ovo* at the developmental stage HH stage 13. The redox status of the sensor was analyzed at HH stage 20. To assess the degree of GRX1-roGFP2 oxidation, transverse sections of St 20 chick spinal cords were imaged by confocal microscopy using excitation wave lengths 405 nm (I405) and 488 nm (I488). Autofluorescence (AF) in I405 channel was separated Grx1-roGFP2 signal by linear unmixing based on the emission spectra and represented in *red* and *green*, respectively. DIC, Differential interference contrast image. Scale bar, 50 μm.

a

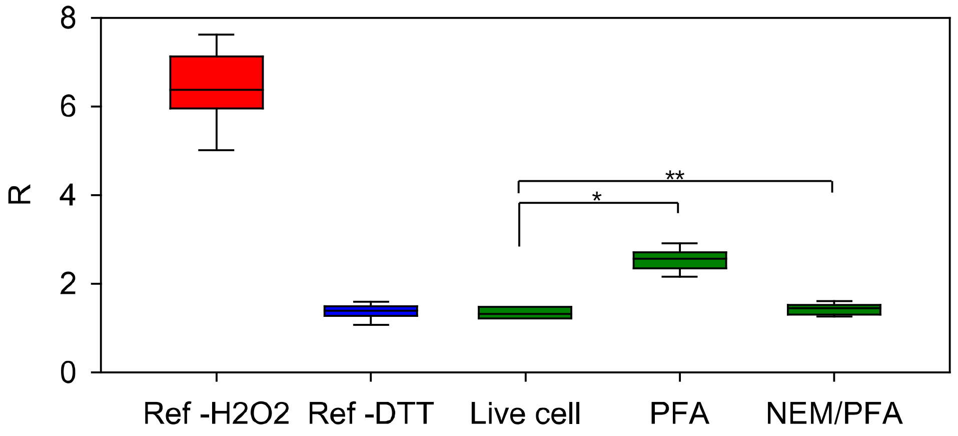

b

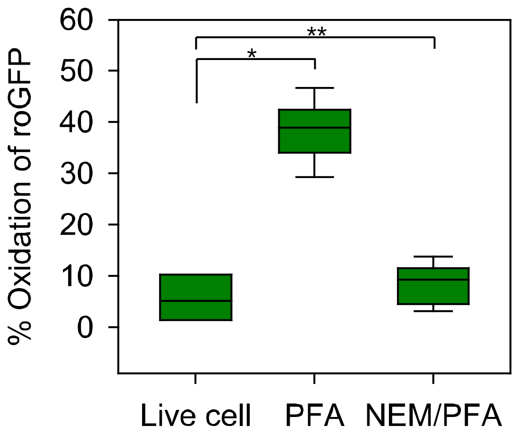

Supplementary Figure 6 | PFA-induced oxidation and protection of Cys by labeling with NEM. (**a**) Grx1-roGFP2 is oxidized by PFA fixation and this can be prevented by pretreatment with N-ethylmaleimide (NEM). To assess the extent of PFA-induced oxidation, HEK293 cells were transfected with pCDNA5/Grx1-roGFP2, fixed with PFA, and the R value was quantified. Live cell without any treatment or fixation (Live cell), fixed cells (PFA), and cells fixed after NEM pretreatment (NEM/PFA) were compared. In addition, live cells were treated with 0.5 mM H_2O_2 or 2 mM DTT for measurement of oxidized or fully reduced sensor. The lower and upper boundaries of the box indicates 25th and 75th percentiles, while whiskers below and above the box represent 10th and 90th percentiles. A horizontal line within the box marks the median. mean±SD; H_2O_2 (6.47±0.92, n=32), DTT (1.37±0.18, n=30), Live cell (1.36±0.16, n=8), PFA (2.53±0.26, n=38), NEM/PFA (1.44±0.17, n=37). *significant at P<0.001. **Not significant at P=0.134. (**b**) R values in *A* were converted to degree of sensor oxidation (OxD) using Hanson's equation. Without pre-treatment, PFA fixation resulted in oxidation of 38% of the sensor. Oxidation was completely blocked by pretreatment with NEM. mean±SD; 6.2%±4.9 point (Live cell), 38%±6.1 point (PFA), 8.2%±5.2 point (NEM/PFA). *significant at P<0.001. **not significant at P=0.33.

a

$$I488_{red} \propto E \cdot (1 - OxD)$$

$$I488_{ox} \propto \frac{E \cdot OxD}{I488min/_{I488max}}$$

$$I488 = I488_{red} + I488_{ox}$$

$$= E \cdot (1 - OxD) + \frac{E \cdot OxD}{I488_{min}/_{I488_{max}}}$$

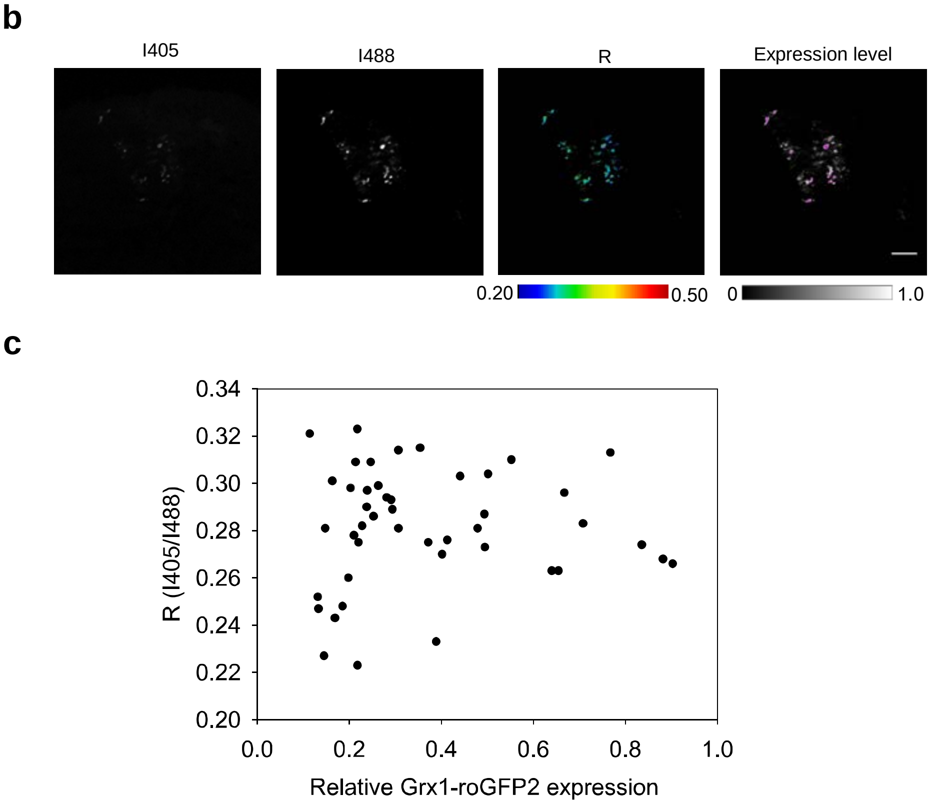

Supplementary Figure 7 | The redox status of Grx1-roGFP2 is independent of expression level. (**a**) Expression level of Grx1-roGFP2 (E) is defined as a function of I488 and OxD. I488 constitutes of contributions from reduced ($I488_{red}$) and oxidized ($I488_{ox}$) forms of Grx1-roGFP2 as shown in the equation. (**b**) The false-color image depicts the expression level of Grx1-roGFP2 calculated from the equations in *a*. Pixels with I488>115 are masked in purple. Bar 50 μm. (**c**) Expression levels and R values in *b* were quantitated for multiple areas (n=45) and plotted. There is no significant relationship between these values; Pearson's product-moment coefficient ρ=0.0427, P=0.78.

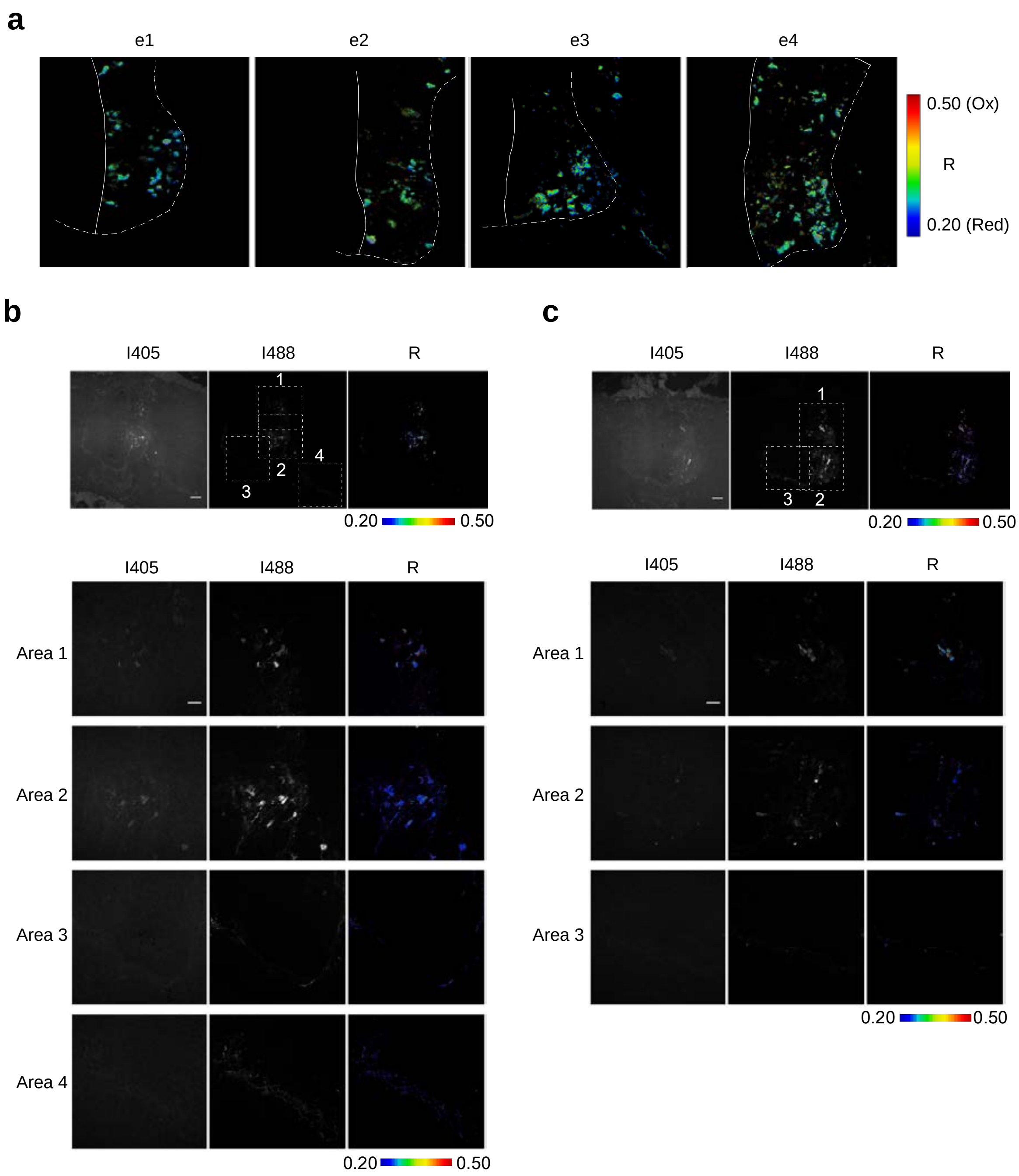

Supplementary Figure 8 | Spatiotemporal redox pattern of cytosolic glutathione within a spinal cord. (**a**) Slices from four different embryos (e1-e4) showed similar trend of redox variation within spinal cords. R value is shown in pseudo-color scale with a range from 0.20 to 0.50 corresponding to 0% to 29% OxD. Regions with saturated I488 signal were masked in gray. (**b**) Sections of HH stage 23 chick spinal cord were similarly analyzed. Four different regions (Area 1-4) were analyzed at higher magnification. The same slide was imaged at different magnification and shown in the main figure 3d. Selected areas are indicated by squares. Bar, 20 μm. (**c**) Another section from the same embryo.

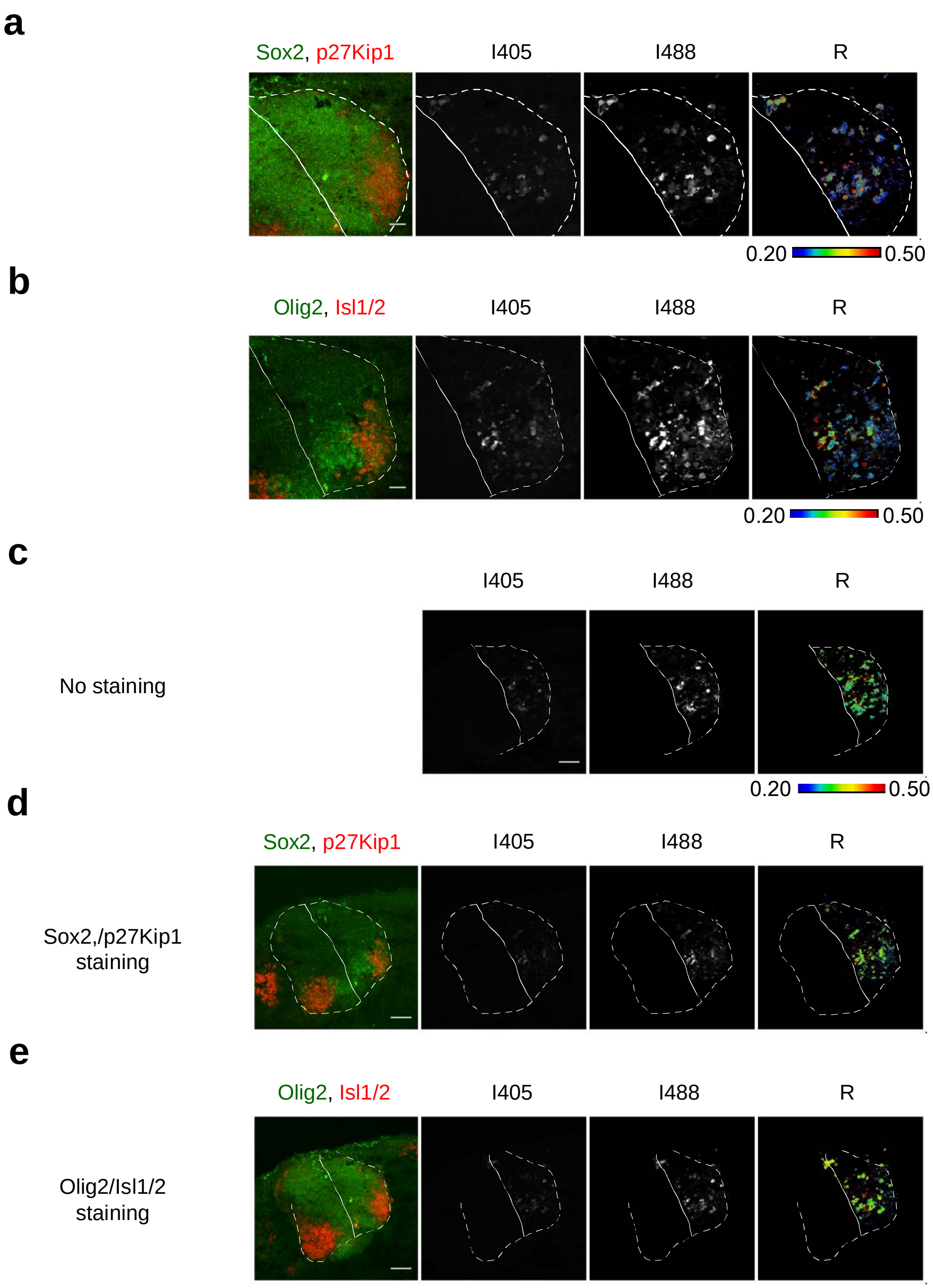

Supplementary Figure 9 | Analyses of Grx1-roGFP2 using immunostained slides. (**a, b**) Immunostained sections were imaged at higher magnification for quantitative analysis. Scale bar, 20 μm. Gain levels were set to avoid signal saturation. Scale bar, 20 μm. (**c-e**) The redox pattern of Grx1-roGFP2 is not affected by immunostaining. Nonstained and immunostained samples show oveall similar trend of redox variations characterized by a shallow gradient from medial to marginal zones. Scale bars, 50 μm.

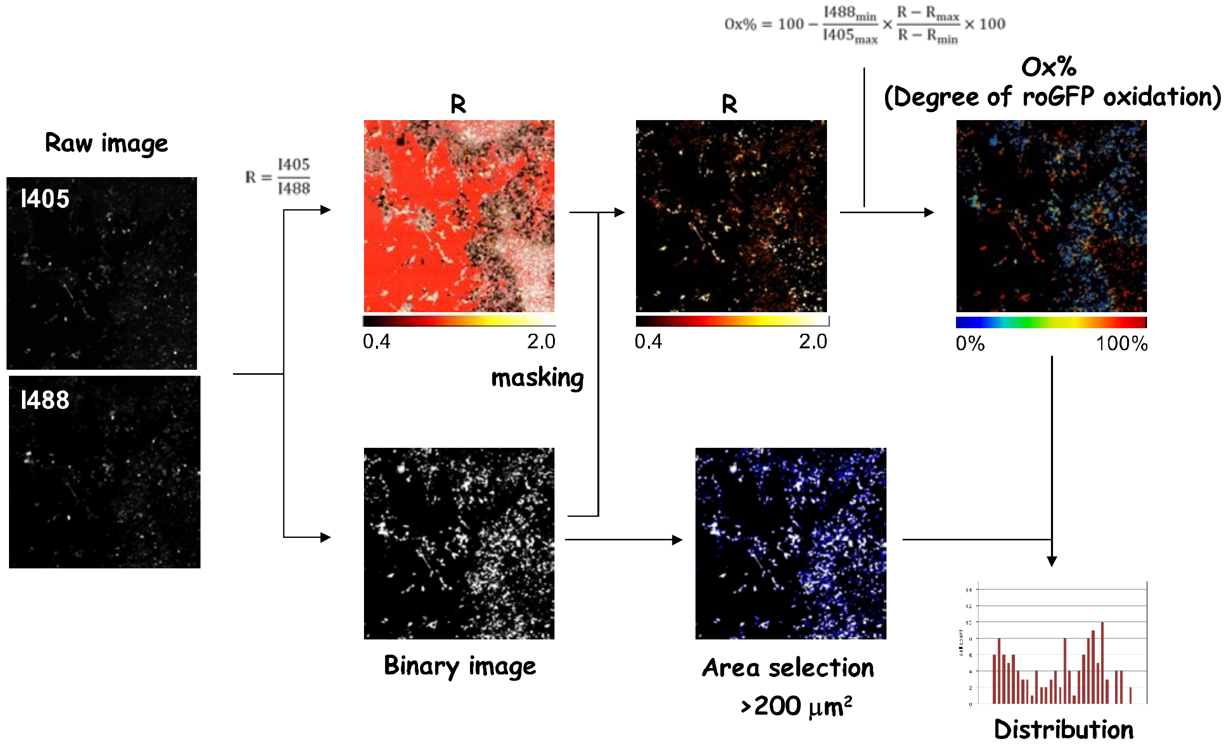

Supplementary Figure 10 | Cell population analysis. Live cells expressing roGFP were imaged for I405 and I488 (8-bit). Areas with dimensions 1125 μm × 1125 μm were imaged by tiling scan (25 images with an object field of 225 μm × 225 μm for each image). Ratiometric image (R) was generated by dividing I405 by I488. Mask binary image was generated from I488 using the threshold of 10 and used for masking background on R image. To exclude pixels with saturated I405 or I488, upper threshold was also set (200). Hanson's equation was then applied to R image for generating OxD image. The same binary image was also used to make area selections. From the all selected areas, areas smaller than 50 μm^2 were excluded from the statistics. Areas with cell aggregation fails were excluded from the analysis using upper threshold of 2,500 μm^2.

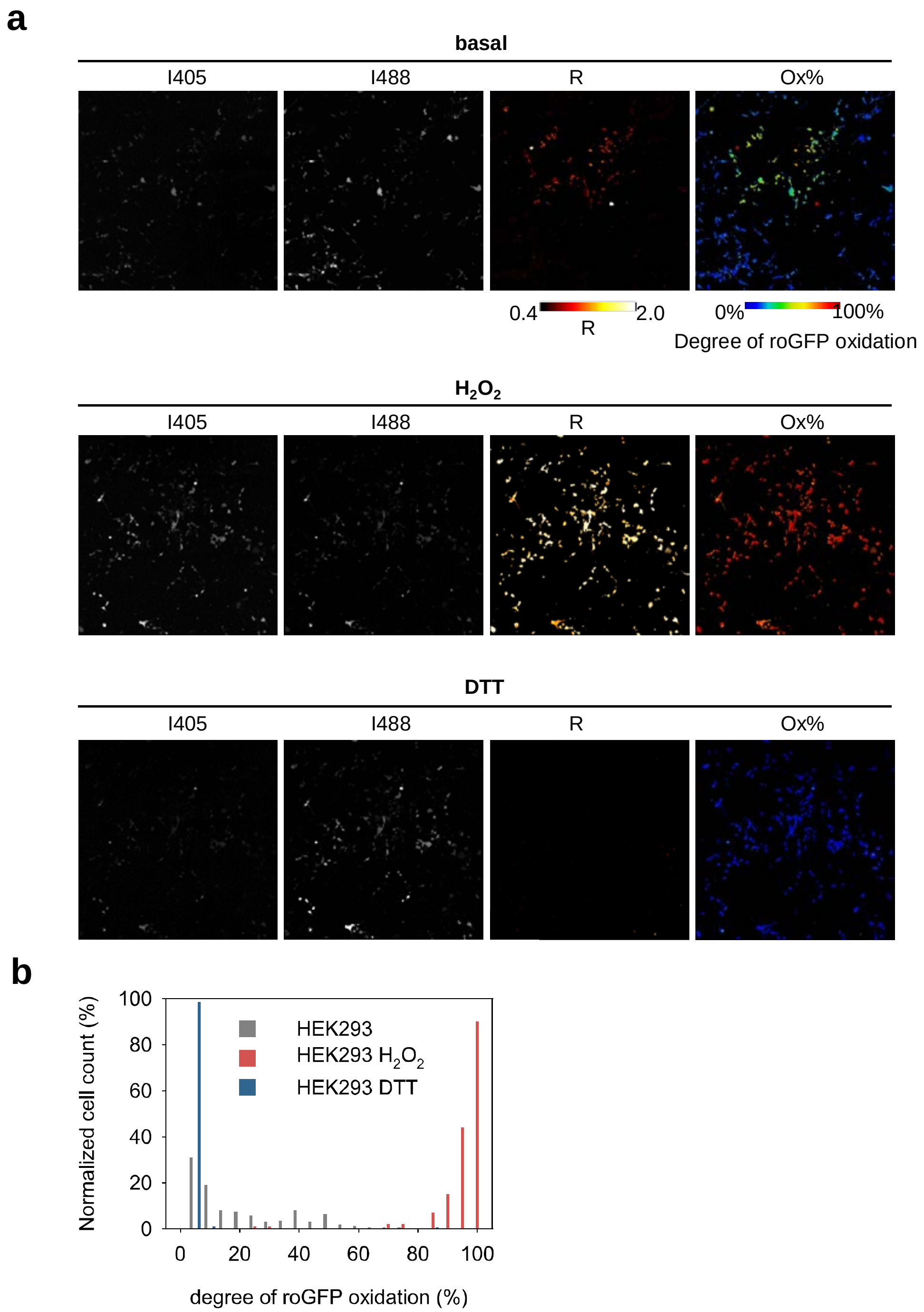

Supplementary Figures 11 | Glutathione redox balance in HEK293 cells. (**a**) The redox assay using roGFP was applied to cell population analysis. HEK293 cells were transfected with pCDN5/Grx1-roGFP2. Imaging was performed on 1125 μm × 1125 μm area. For oxidized and reduced controls, cells were sequentially treated with 500 μM H_2O_2 and 2 mM DTT. Degree of roGFP oxidation (OxD) was estimated from R value using Hanson's equation[26] and rendered in pseudo-color scale. Bar, 200 μm. (**b**) Histogram for images shown in *a*. n=175, 181, 201 for basal, H_2O_2, DTT conditions, respectively.

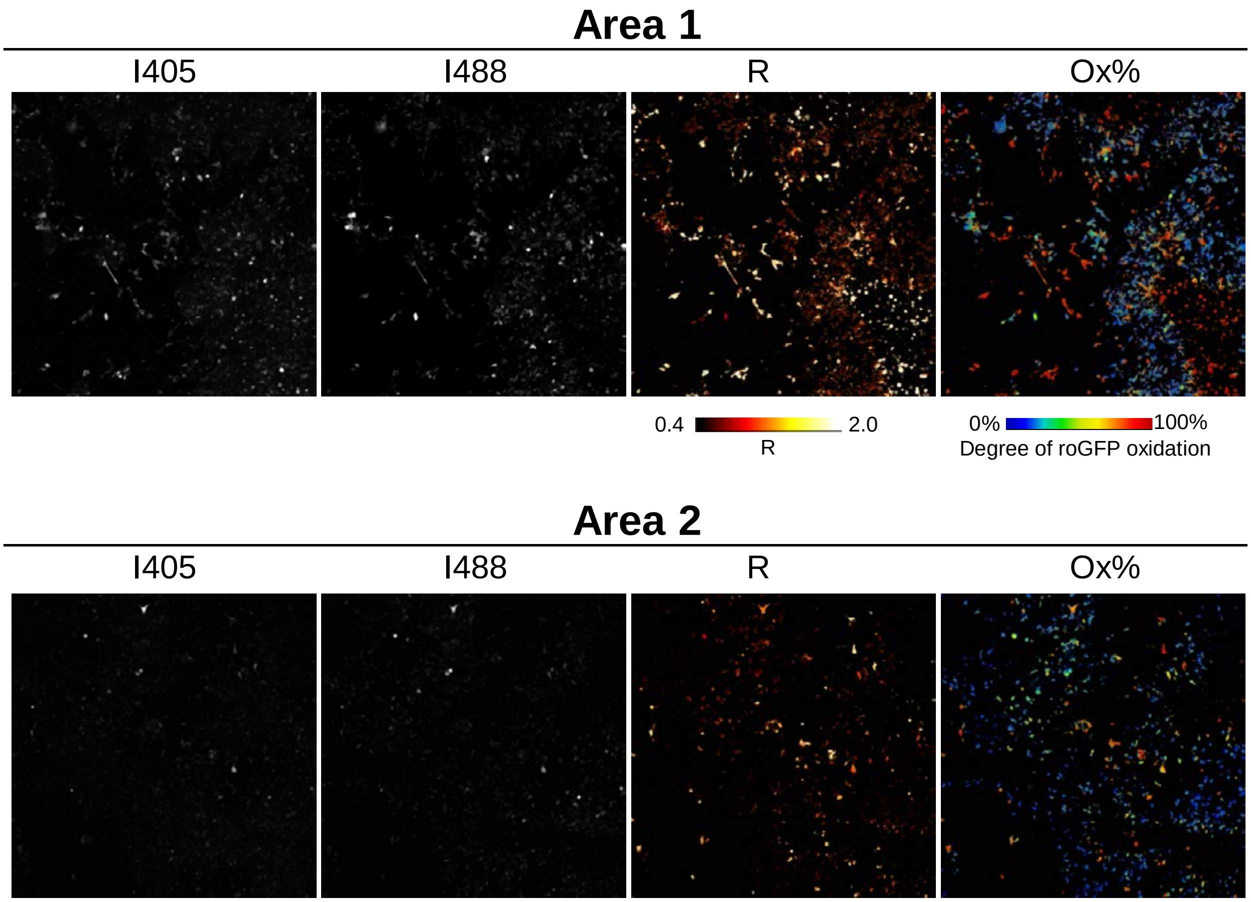

Supplementary Figures 12| Glutathione redox balance in non-differentiated SH-SY5Y cells. Images used for generating the main figures 4c and d.

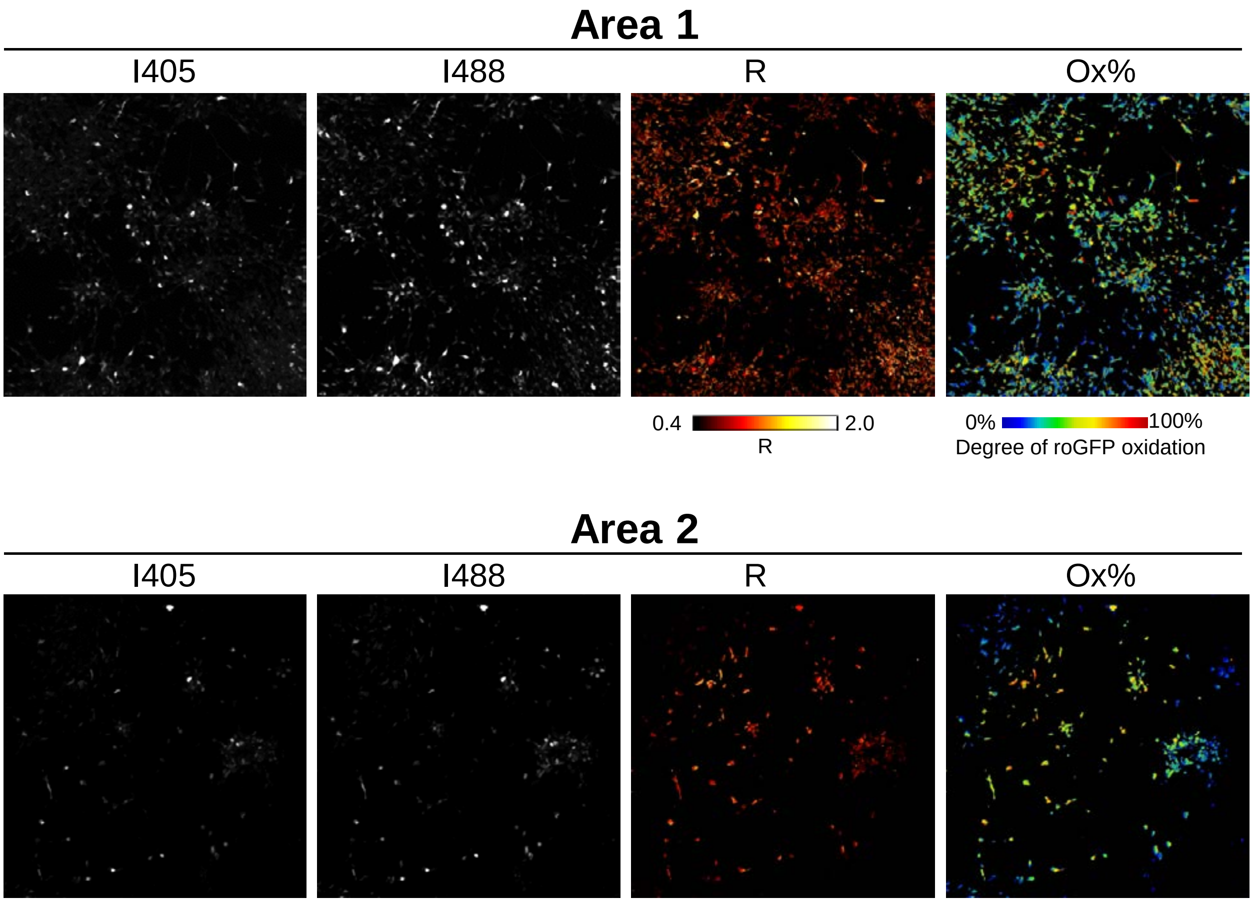

Supplementary Figures 13| Glutathione redox balance in retinoic acid-treated SH-SY5Y cells. Images used for generating the main figures 4c-f.

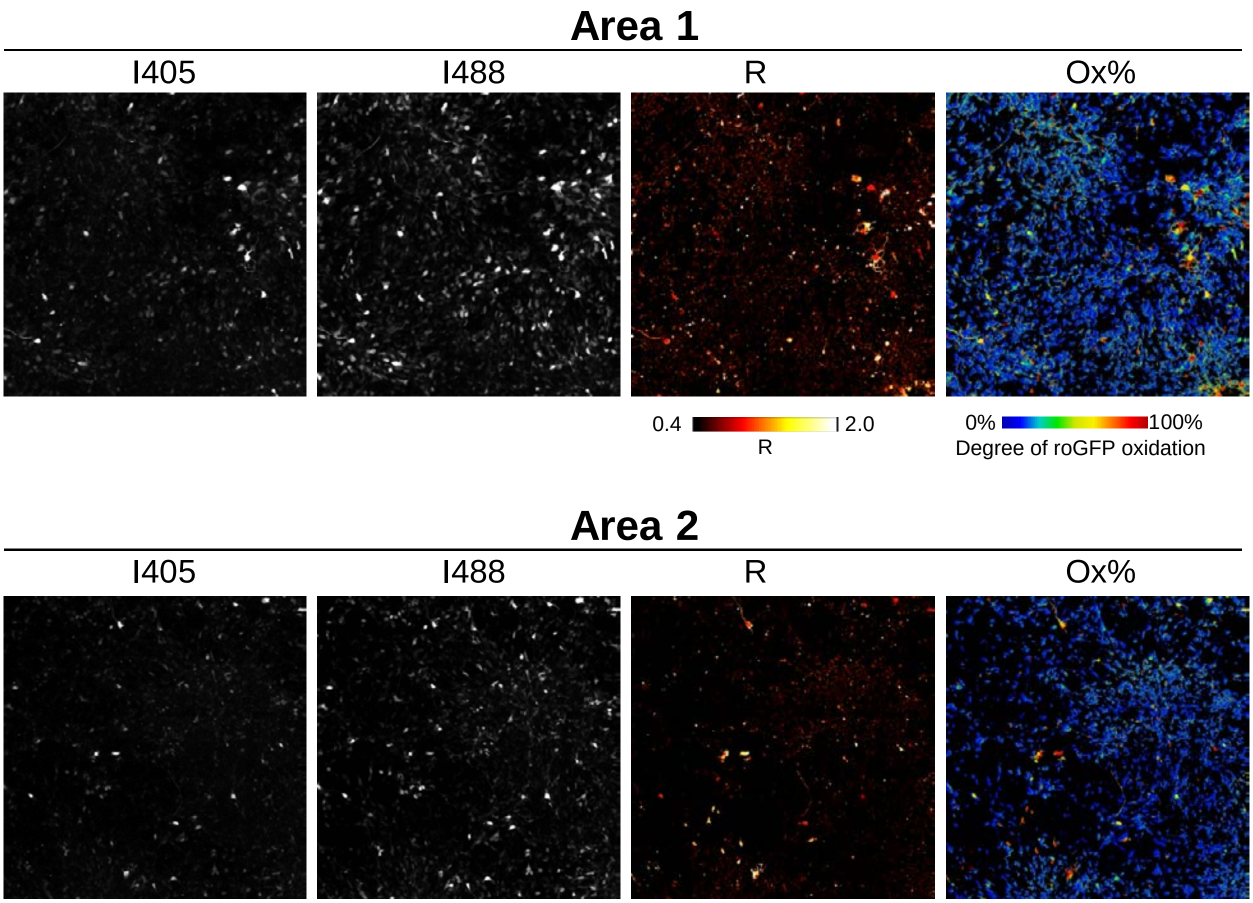

Supplementary Figures 14| Glutathione redox balance in differentiated SH-SY5Y cells. Images used for generating the main figures 4e and f.

a

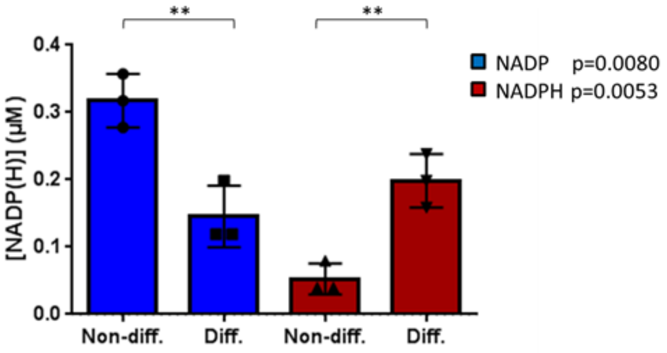

b

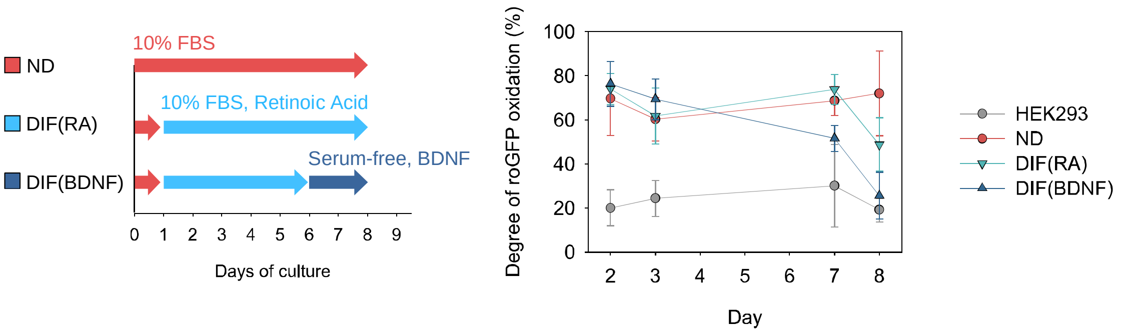

Supplementary Figure 15 | Glutathione and NADPH redox balance in SH-SY5Y cells changes over time and depend on treatment with differentiation inducers. (**a**) NADP$^+$ and NADPH levels in non-differentiated and differentiated SH-SY5Y cells were measured (*n*=3). The same amount of cells (10^5 cells per sample) were lysed in equal volumes for all samples and NADP(H) concentration in each sample was determined (see methods). Squares, triangles, and circles indicate each data point. **differences are significant at *P*<0.01, Student's *t*-test. (**b**) Cells were cultured in 3 distinct courses of incubation (*left*, ND, RA, and BDNF) and roGFP analysis was performed at different time points (*right*). OxD values were calculated from R values. n=20 for each condition/time point. For comparison as well as control, HEK293 cells were analyzed simultaneously.

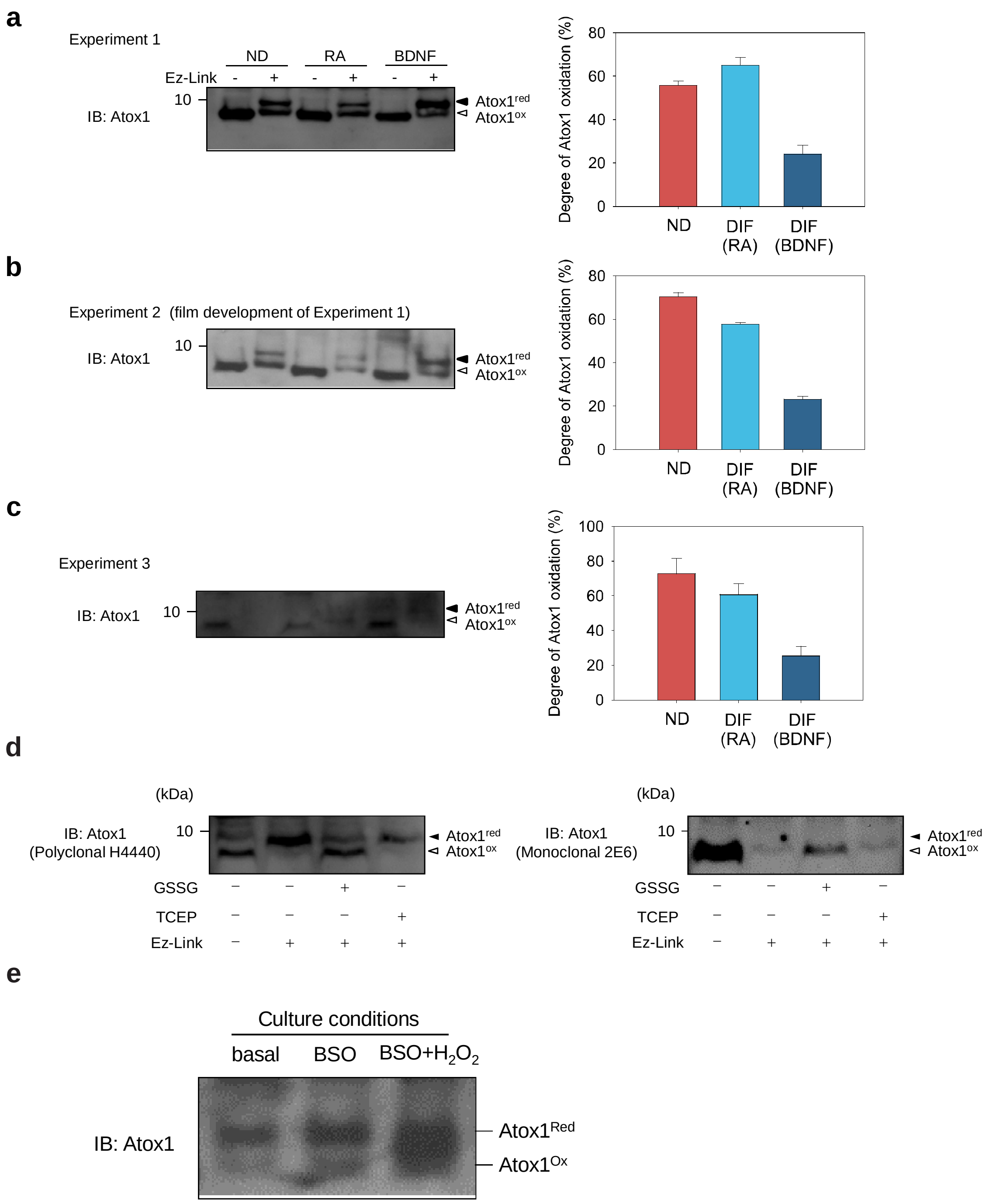

Supplementary Figure 16 | Redox status of Atox1 in SH-SY5Y cells at various differentiation stages. (**a-c**) Three independent results of Cys-labeling experiments for determining the redox status of Atox1 in SH-SY5Y cells. Representative image *a* was shown in the main figure 5b. Based on the results of densitometry from these 3 independent analyses, mean of mean values were calculated and shown in the main figure 5b. (**d**) Comparison of the results for two different antibodies; rabbit antisera raised against human Atox1 (polyclonal H4440, left) and mouse monoclonal anti-Atox1 antibody (clone 2E6, right). Cell lysates

(HEK293) were pretreated with either 1 mM reducing reagent tris(2-carboxyethyl)phosphine (TCEP) or 1 mM oxidized glutathione (GSSG) for 30 min where indicated. Labeled proteins were separated on 15% Tricine gel. The higher and lower bands represent reduced and oxidized forms of Atox1, respectively. Antibody 2E6 did not detect fully labeled Atox1 most likely due to masking of the epitope by labeling. For this property, labeled Atox1 was detected using antisera H4440 in all other experiments.). (**e**) Atox1 is oxidized by combined treatment with BSO and hydrogen peroxide. Differentiated cells (DIF BDNF) were treated with 0.5 mM buthioninesulfoxide (BSO) for 6 h followed by adding 100 μM hydrogen peroxide. After 3 h incubation, redox status of Atox1 was determined.

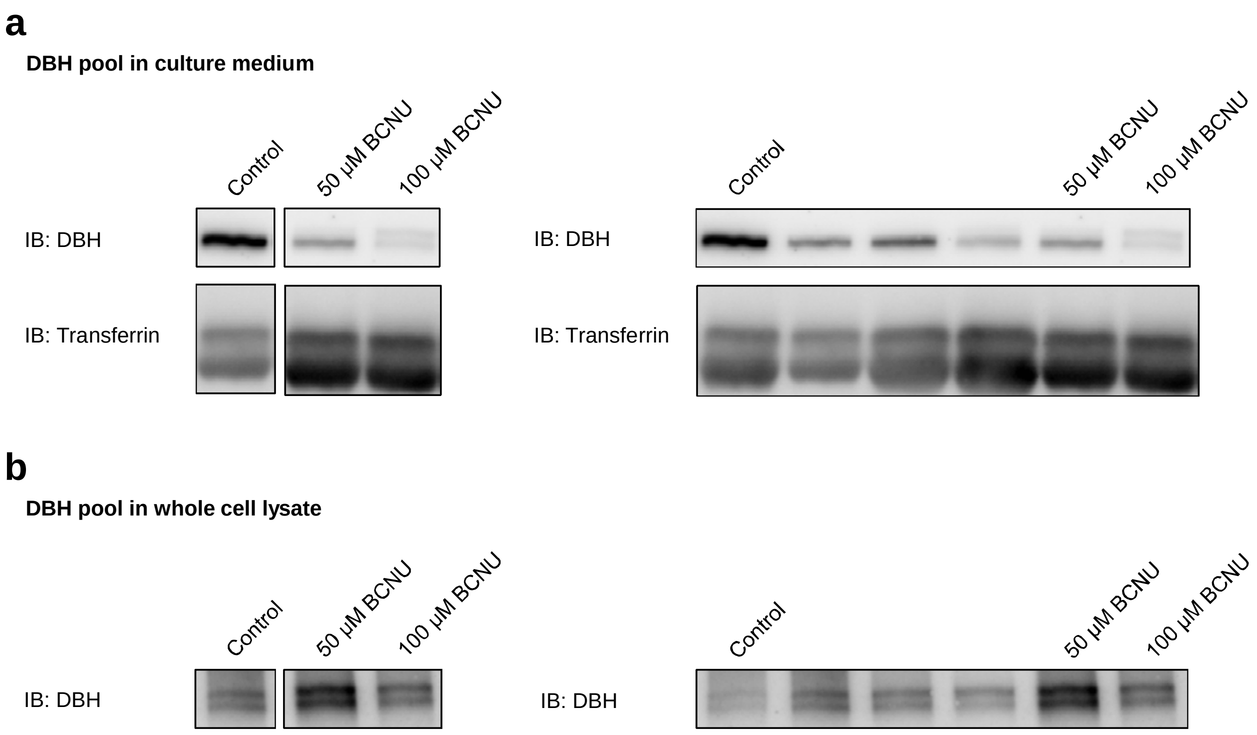

Supplementary Figure 17 | Glutathione oxidation resulted in decrease of DBH secretion. (**a**) Secreted DBH pool was decreased upon glutathione oxidation. SH-SY5Y cells were semi-differentiated by retinoic acid (see method in the main text) and treated with 50 μM or 100 μM BCNU to induce glutathione oxidation. Equal volume of culture medium was harvested, concentrated by ultrafiltration, and analyzed for DBH protein level by western blotting. BCNU treatment led to decrease in secreted DBH protein level. For comparison, secreted transferrin was quantified, which is rather slightly increased. The original contiguous image is shown in the right. (**b**) Impaired secretion of DBH in BCNU-treated cells led to accumulation of intracellular pool of DBH. Equal protein amount of whole cell lysate (15 μg per lane) was loaded. The original contiguous image is shown in the right.

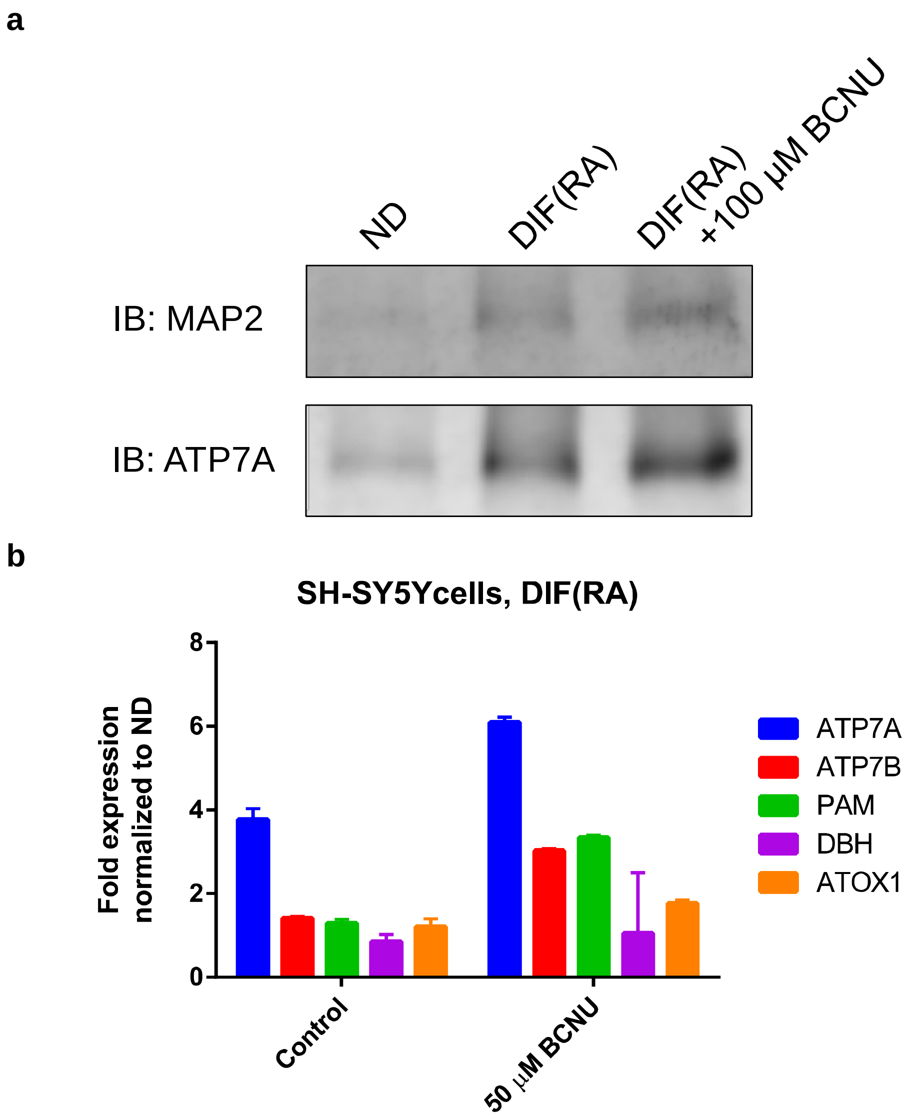

Supplementary Figure 18 | Glutathione oxidation did not affect mRNA levels of ATP7A and Atox1. (**a**) Retinoic acid (RA) treatment led to increase of ATP7A and MAP2 (a marker of neuronal differentiation) protein levels regardless of the redox balance of cellular glutathione. SH-SY5Y cells were semi-differentiated by retinoic acid (see method in the main text) and treated with 100 μM BCNU to induce glutathione oxidation. Equal protein amount of whole cell lysate (15 μg per lane) was loaded. (**b**) Glutathione oxidation did not affect RA-dependent upregulations of ATP7A, ATP7B, PAM, and Atox1. Cells were prepared as described in A and mRNA levels of indicated genes were quantified by qPCR (see methods in the main text). Values are normalized to non-differentiated cells. Sequences of DNA primers are shown in Supplementary Figure 19.

a

Oligonucleotides used for subcloning

Oligo name	Sequence
Atox1_f_NheI	5′-TTGCTAGCATGCCGAAGCACGAGTTCTCTGTG-3′
Atox1_r_EcoR	5′-TTGAATTCTCCTCCATCAAGGCCAAGGTAGGAAACAG-3′
CtermEGFP_f_EcoRI	5′-AAGAATTCATGGTGAGCAAGGGCGAGGAGCTGTTCACC-3′
CtermEGFP_r_NotI	5′-AAGCGGCCGCTTACTTGTACAGCTCGTCCATGCCGAGAGTGATC-3′

Oligonucleotides used for qPCR

Oligo name	Target gene	Sequence
hGAPDH_f	GAPDH	5′-CATCAATGGAAATCCCATCA-3′
hGAPDH_r	GAPDH	5′-GACTCCACGACGTACTCAGC-3′
hAtox1_f	Atox1	5′-CTCTCGGGTCCTCAATAAGC-3′
hAtox1_r	Atox1	5′-GTTGCAAGCAGAGTGTCCAT-3′
hATP7A_f	ATP7A	5′-ATTGATGACATGGGCTTTGA-3′
hATP7A_r	ATP7A	5′-GCAATGTGCTTTGGATATGG-3′
hATP7B_f	ATP7B	5′-AGGAGCCCTGTGACATTCTT-3′
hATP7B_r	ATP7B	5′-TTGCTCTTTGCCAAGTGTTC-3′
hPAM_f	PAM	5′-GGGCATTACAATCCTGTCCT-3′
hPAM_r	PAM	5′-CAGTGGAAAGATTGCCTGAA-3′
hHEP_f	HEP	5′-TTTCCTGAGCAACAAGGATG-3′
hHEP_r	HEP	5′-TAAGTGGACCCAAGATTCCC-3′
hCCS_f	CCS	5′-CAACAGCTGTGGGAATCACT-3′
hCCS_r	CCS	5′-AGCTGCTCATCCTCCATTCT-3′
hSOD1_f	SOD1	5′-TGAAGAGAGGCATGTTGGAG-3′
hSOD1_r	SOD1	5′-ATGATGCAATGGTCTCCTGA-3′
hCOX17_f	COX17	5′-CGATGCGTGTATCATCGAG-3′
hCOX17_r	COX17	5′-ACACAGCAGACCACCATTTC-3′
hCOX1_f	COX1	5′-CGATGCATACACCACATGAA-3′
hCOX1_r	COX1	5′-TCCAGGTTTATGGAGGGTTC-3′
hCTR1_f	CTR1	5′-GACCAAATGGAACCATCCTT-3′
hCTR1_r	CTR1	5′-ATGACCACCTGGATGATGTG-3′
hCTR2_f	CTR2	5′-TCAGATTCATTCCCTGTTGG-3′
hCTR2_r	CTR2	5′-GCATGATGAAGTAGCCGATG-3′

b

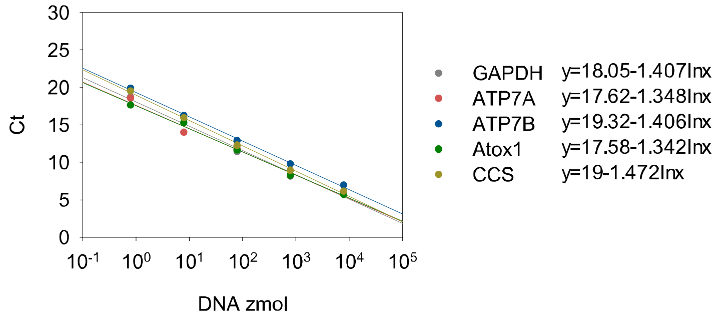

Supplementary Figure 19 | Oligonucleotides used in the study. (**a**) All qPCR primers are designed to target human cDNA. (**b**) Standard curves for absolute quantitation of mRNA by qPCR. Amplicons of GAPDH, ATP7A, ATP7B, Atox1, and CCS were subcloned into pCR2.1-TOPO vector and purified plasmids were used as standards for qPCR. DNA concentrations were determined by absorbance at 260 nm. As shown in *b*, amplification efficiency close to 2.0 (the theoretical optimum) was obtained for all the primer sets.
